# Changes in air quality during COVID-19 ‘lockdown’ in the United Kingdom^☆^

**DOI:** 10.1016/j.envpol.2020.116011

**Published:** 2021-03-01

**Authors:** Calvin Jephcote, Anna L. Hansell, Kathryn Adams, John Gulliver

**Affiliations:** a Centre for Environmental Health and Sustainability, University of Leicester, United Kingdom; b NIHR HPRU Centre for Environmental Exposures and Health, University of Leicester, United Kingdom

**Keywords:** Air quality, Air pollution intervention, COVID-19, NO2, PM2.5, O3

## Abstract

The UK implemented a lockdown in Spring (2020) to curtail the person-to-person transmission of the SARS-CoV-2 virus. Measures restricted movements to one outing per day for exercise and shopping, otherwise most people were restricted to their dwelling except for key workers (e.g. medical, supermarkets, and transport). In this study, we quantified changes to air quality across the United Kingdom from 30/03/2020 to 03/05/2020 (weeks 14–18), the period of most stringent travel restrictions. Daily pollutant measurements of NO_2_, O_3_ and PM_2.5_ from the national network of monitoring sites during this period were compared with measurements over the same period during 2017–19. Comparisons were also made with predicted concentrations for the 2020 period from business-as-usual (BAU) modelling, where the contributions of normal anthropogenic activities were estimated under the observed meteorological conditions. During the lockdown study period there was a 69% reduction in traffic overall (74% reduction in light and 35% in heavy vehicles). Measurements from 129 monitoring stations, identified mean reductions in NO_2_ of 38.3% (−8.8 μg/m^3^) and PM_2.5_ of 16.5% (−2.2 μg/m^3^). Improvements in NO_2_ and PM_2.5_ were largest at urban traffic sites and more modest at background locations where a large proportion of the population live. In contrast, O_3_ concentrations on average increased by 7.6% (+4.8 μg/m^3^) with the largest increases at roadside sites due to reductions in local emissions of NO. A lack of VOC monitoring limited our capacity to interpret changes in O_3_ at urban background locations. BAU models predicted comparable NO_2_ reductions and O_3_ gains, although PM_2.5_ episodes would have been more prominent without lockdown. Results demonstrate the relatively modest contribution of traffic to air quality, suggesting that sustained improvements in air quality require actions across various sectors, including working with international and European initiatives on long-range transport air pollutants, especially PM_2.5_ and O_3_.

## Highlights

• Monthly-average daily traffic counts in April-2020 fell by 69% compared to April-2019.
• NO_2_ and PM_2.5_ concentrations fell respectively by 38.3% (8.8 μg/m^3^) and 16.5% (2.2 μg/m^3^).
• O_3_ increased by 7.6% overall as the atmospheric chemistry changed.
• NO_2_ and PM_2.5_ improvements were largest at urban traffic sites.
• Weather conditions contributed to particulate episodes that were seen despite traffic reductions.

## Graphical abstract

**Figure:**
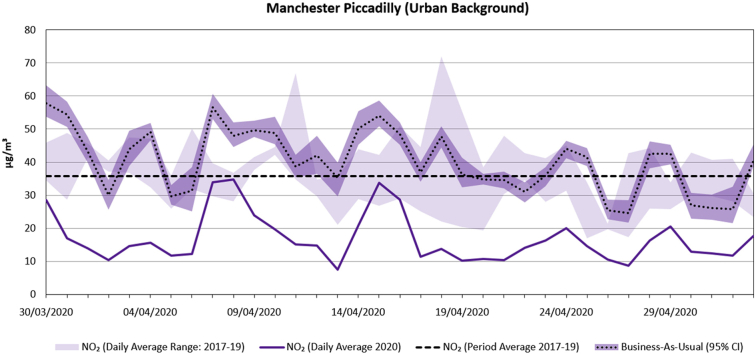

## 1 Introduction

The first human cases of a severe acute respiratory syndrome coronavirus (SARS-CoV-2), referred to as COVID-19, were detected in Wuhan City, China, in December 2019. The virus spread rapidly in early 2020, initially from China to Italy and Europe where lockdowns were introduced restricting travel and industrial activity to control spread. These resulted in marked improvements in air quality with the dramatic extent of this apparent in international satellite imagery (Menut et al., 2020; NASA 2020; Shi and Brasseur, 2020).

The UK confirmed its earliest cases on 31/01/2020, with rapid increase in cases to >5000 by 20/03/2020 resulting in a national lockdown starting on 24/03/2020 (UK Government, 2020). Restrictions introduced included; (a) people to stay at home, except for limited purposes; (b) closing of all non-essential businesses (including retail and hospitality sectors) and public venues (including community centres, libraries, and places of worship); and (c) stopping all gatherings of more than two people in public (Johnson 2020). The media widely reported falls in air pollution of 40%–60% in early April (Khoo, 2020; Telegraph Reporters., 2020). At the same time the UK Department for Environment Food & Rural Affairs (Defra) was warning of air pollution episodes with the Daily Air Quality Index reaching the highest level on its 10-point measurement scale.

This paper investigates these apparently contradictory statements, looking at air pollutant concentrations at monitoring sites across the UK for nitrogen dioxide (NO_2_), nitrogen oxides (NO_X_), ozone (O_3_), fine particles (PM_2.5_), and volatile organic compounds (VOCs). We compared measurement data during the lockdown study period to (a) measurements in previous years, and (b) modelled concentrations expected in a business-as-usual scenario, where sources of emission continue to operate as normal under the observed meteorological conditions (i.e. if the lockdown measures had not been implemented). We also looked at information on numbers of light and heavy vehicles during lockdown, to assess how the UK travel-restrictions influenced air quality. Such information from a real-world natural ‘experiment’ can help in understanding the likely impact of prospective intervention-based policies that target vehicle fleet volume and composition.

## 2 Materials and methods

### 2.1 Air quality measurements

The UK has a network of 170 active monitoring stations in urban and rural areas that form the Automatic Urban and Rural Network (AURN), providing measurements that primarily measure pollutants generated (indirectly or directly) from vehicular, industrial and domestic emissions, in accordance with European Parliament (2008) Directive 2008/50/EC.

Hourly measurements of gaseous and particulate pollutants recorded by AURN sites, were accessed in R-Statistical Software via the openair v2.7 application programming interface (API). Measurements of NO_2_, NO_X_, O_3_, and PM_2.5_ were extracted from all monitoring sites with a 95% or above hourly capture rate across weeks 14–18 of 2020. This covered a 5-week period of the most stringent restrictions on population movements during an outbreak of COVID-19 in the UK, where employers in non-essential sectors facilitated homeworking or granted temporary leaves of absence. The start date was one week after the UK government’s imposed travel restrictions on 23/03/2020, allowing for a transition in travel behaviour. The study end date was 5-days before a public holiday, which was anecdotally linked with widespread travel breaches. Daily average concentrations were only calculated for days with a minimum hourly capture rate of 75%.

For comparison, air quality measurements at the selected sites were obtained across weeks 14–18 of 2017, 2018, and 2019. AURN sites were only included in the final analysis if they also reported a 95% or above hourly capture rate over this 3-year period. As before, daily average concentrations were calculated for days with a minimum hourly capture rate of 75%. Table S1 provides a breakdown of the AURN sites included in this analysis, by type and the availability of pollutant measurements.

Defra (Department for environment, food and rural affairs) uses a three-fold classification to separate monitoring stations into urban, suburban, or rural locations. These locations are then sub-divided into traffic, industrial and background sites, indicative of the predominant source of pollution in the area. There are six types of measurement site in the AURN: urban traffic (UT), urban industrial (UI), urban background (UB), suburban-industrial (SI), suburban background (SB), and rural background (RB). Additional information on the classification of monitoring stations by site type is provided in Table S2**.**

Air quality ‘improvements’ were quantified at each site by reporting the (a) number of days above, and (b) percentage changes on the mean daily-averaged pollutant concentrations measured in weeks 14–18 of 2017, 2018, and 2019. The results were also summarised by site type and region, with further interpretation only provided for groups containing three of more sites to limit representative bias.

### 2.2 Traffic counts

Monthly-average daily traffic (MADT) counts on A-Roads and Motorways were obtained from Highways England in R-Statistical Software via the webTRISr v0.2 API. 5898 sites located on major road links across England, returned traffic counts for the months of January to May, in 2019 and 2020. Changes (%) on the previous year’s MADT count were reported for individual months, summarised by region and vehicle type. This covers the build-up to lockdown, and two months of lockdown with varying degrees of compliance.

### 2.3 Meteorological measurements

Hourly measurements of wind-speed (m/s) and wind-direction (°) were directly recorded at the AURN measurement sites. Hourly measurements of other meteorological parameters recorded at UK Met Office stations, were accessed in R-Statistical Software via the worldmet v0.9 API. This measurement data is provided by the National Oceanic and Atmospheric Administration’s (NOAA) global integrated surface database of hourly climatological observations, subject to ratification and quality assurance processes (Lott 2004).

We obtained hourly air temperature (°C), atmospheric pressure (mb), cloud-ceiling height (m), dew point (°C), and relative humidity (%) for each AURN site using measurements from the nearest Met Office station with a 90% or above data capture on each parameter (66% threshold used for cloud cover). Missing values were imputed using an enhanced polynomial interpolation method, which minimises overshooting and spurious oscillations in time-series data (Stineman, 1980). On average 1.2%, 1.6%, 1.3%, 1.3% and 17.6% of the hourly measurements for air temperature, atmospheric pressure, dew point, relative humidity, and cloud-ceiling height respectively were imputed at each site. The AURN sites of interest and Met Office stations are, on average, separated by a distance of 38 km (interquartile range of 20 km–50 km).

### 2.4 Business-as-usual scenario modelling

Boosted Regression Trees (BRT) models were used to estimate daily-average air pollution concentrations following a business-as-usual scenario (BAU). In this scenario, local emission sources continue to operate as normal under the meteorological conditions observed in weeks 14–18 of 2020. These forecasts allow for a direct comparison with day-to-day measurement data.

BRT models were constructed in R-Statistical Software, using the ‘gbm’ v2.1.8 package. For each UK-AURN site, 75% of the hourly meteorological and pollutant measurements recorded across the period of 01/01/2017 to 31/12/2019 were used to train the BRT models, with 25% of the data used to cross-validate the machine learning approach. AURN sites were only included in this analysis where local source contributions follow a predictable pattern for a given set of meteorological conditions and temporal structures, as indicated by a cross-validation R^2^ ≥ 0.75. Further details relating to the development and verification of these models are provided in Tables S3–S4.

This forecasting approach was used for verification purposes, to ensure that any findings from the comparison of measured data have not been skewed by unusual meteorological conditions. Although comparisons of measurement data are only subject to instrument error, they are to an extent temporally limited by their comparison to a pollutant threshold, which is representative of meteorologically averaged conditions in a recurring 5-week period across 2017–19. In contrast, BAU forecasts allow for a day-to-day comparison with measurement data under identical meteorological conditions for each hour of the day, but at the expense of modelling uncertainty.

## 3 Results

### 3.1 Environmental measurements

On 23/03/2020, the UK government implemented lockdown restrictions on population mobility. Compared to 01/03/2020, national levels of road transport levels had decreased to 81% by 20/03/2020, sharply falling to 49% on 24/03/2020, and finally settling at around 36% on 30/04/2020 (COBRA 2020). Monthly-average daily traffic (MADT) counts on English A-roads and motorways in April 2020 were down by 69% compared with April 2019, with a 74% reduction in light and a 35% reduction in heavy (coaches and HGVs) vehicle counts (see Table S5). This pattern was similar across all regions. A gradual increase in the number of passenger vehicles was observed in the month of May, with light and heavy vehicle counts in England recorded at 60% and 30% below May 2019 levels, respectively.

In terms of meteorological parameters, much of April 2020 was fine and settled, with a high-pressure system seeing light easterly winds and benign conditions. The provisional UK mean temperature of 9.1 °C was 1.7 °C above the 1981–2010 long-term average, making it the fifth warmest April since 1884 (Met Office 2020). It was ranked as the sunniest April since 1929, experiencing 151% of the average sunshine and only 40% of the average rainfall, most of which fell on the last four days of the month (Met Office 2020). Weekly-average meteorological profiles at urban background sites, recorded consistently low wind-speeds (<4 m/s) in southern and central England, rising to 5–6 m/s in Scotland and Wales (see Table S6). Easterly winds (45–135°) were typically reported throughout weeks 15–17 in England and Wales, returning to a prevailing south-westerly in week 18. These meteorological conditions are favourable for photochemical reactions and the stagnation of air masses, resulting in a build-up of local emissions.

In total, 129 AURN sites were included in this study. Reductions in nitrogen dioxide (NO_2_) concentrations were reported across the UK, regardless of the measurement stations site type (Table 1). Compared to measurements in the previous three years, NO_2_ concentrations at urban traffic sites fell by 47.9% on average. For urban background monitoring sites, we saw reductions in NO_2_ of 36.7% on average. Falls in regions with three or more active urban background monitoring sites ranged from 20.8% in the South West to 44.7% in Scotland (see Table S7 and Figs. S1–S2). Smaller reductions in NO_2_ were observed in suburban and rural areas. NO_2_ decreased on 2017-19 levels by 50.5% at suburban industrial sites with valid measurement data (count = 2), compared to levels falling by 14.8% at suburban background sites (count = 3). This reflects changes in travel patterns involving places of residence and the workplace. NO_x_ concentrations, which include non-oxidised and oxidised nitrogen emissions released by combustion sources, had comparable levels of reduction to NO_2_.

**Table 1:** Summary by site type in England, Scotland, Wales, and Northern Ireland.

Site Type	5-week average daily concentrations, covering April 2020 in μg/m^3^ (AURN count)^a^				% change on the 5-week average daily concentration measured in 2017–19^a^				Absolute change on the 5-week average daily concentration measured in 2017–19 (μg/m^3^)^a^				% days below the 5-week average daily concentration in 2017–19			
	NO_2_	NO_X_	O_3_	PM_2.5_	NO_2_	NO_X_	O_3_	PM_2.5_	NO_2_	NO_X_	O_3_	PM_2.5_	NO_2_	NO_X_	O_3_	PM_2.5_
Rural Background	5.7 (9)	10.1 (11)	73.7 (16)	11.5 (3)	−23.9	+17.5	+0.1	−2.6	−1.8	+1.5	+0.1	−0.3	78.4	73.5	48.9	67.6
Suburban Background	15.0 (3)	18.5 (3)	68.1 (2)	14.5 (2)	−14.8	−18.6	−0.4	−12.0	−2.6	−4.2	−0.3	−2.0	71.4	76.2	52.9	72.9
Urban Background	12.6 (41)	16.6 (41)	66.4 (31)	10.9 (28)	−36.7	−37.8	+7.4	−17.3	−7.3	−10.1	+4.6	−2.3	84.1	85.6	33.7	74.2
Urban Traffic	17.5 (47)	30.0 (47)	57.8 (3)	10.4 (11)	−47.9	−57.3	+34.1	−18.1	−16.1	−40.3	+14.7	−2.3	93.5	95.6	11.4	75.8
Suburban Industrial	10.4 (2)	14.0 (2)	63.3 (1)	–	−50.5	−49.6	+2.9	–	−10.6	−13.8	+1.8	–	98.6	98.6	34.3	–
Urban Industrial	11.0 (5)	13.4 (6)	68.3 (3)	10.5 (3)	−30.6	−33.3	+7.4	−15.3	−4.9	−6.7	+4.7	−1.9	86.2	85.6	36.2	69.5
All Sites	14.1 (107)	21.5 (110)	68.1 (56)	10.9 (47)	−38.3	−38.0	+7.6	−16.5	−8.8	−13.2	4.8	−2.2	87.8	88.6	37.7	73.8

a *Calculations required at least 95% of the daily-average measurements to be available in (a) weeks 14 to 18 of 2020, and (b) weeks 14 to 18 of 2017-19. Days were excluded from the 5-week average calculations, where less than 18-h of measurement data is available*.

Changes in ozone (O_3_) varied markedly by site type. Changes in O_3_ were overall smaller (+7.6%) and more variable, with slight falls in suburban areas (−0.4%) and slight rises in rural (+0.1%) and urban background (+7.4%) areas, related to changes in balance of atmospheric chemistry with differing pollutant emissions. This is reflected in regional changes in O_3_, which fell by 11.0% in Scotland, and rose by 23.0% in London. O_3_ concentrations increased at all roadside sites (count = 3) by an average of 34.1%. O_3_ at the London Marylebone road urban traffic sites was 79.5% above concentrations reported in previous years, while sites in the West Midlands and South West reported increases of approximately 20.6% (see Table S8).

For PM_2.5_ we saw reductions of 16.5% overall, ranging from 12.6% in London to 25.7% in the East Midlands at urban background sites. Again, higher falls were seen in urban compared with suburban and rural areas. Despite the general falls in PM_2.5_, four of 47 sites monitoring sites of this pollutant experienced an overall rise in concentrations. These were all in Southern England and during the warm, still, sunny weather over Easter. They include the rural background sites at Chilbolton (+4.0%) and Rochester Stoke (+5.0%), along with an urban traffic site (+23.2%) near to Plymouth, and an urban background site in Plymouth (+40.5%).

**Fig. 1:**
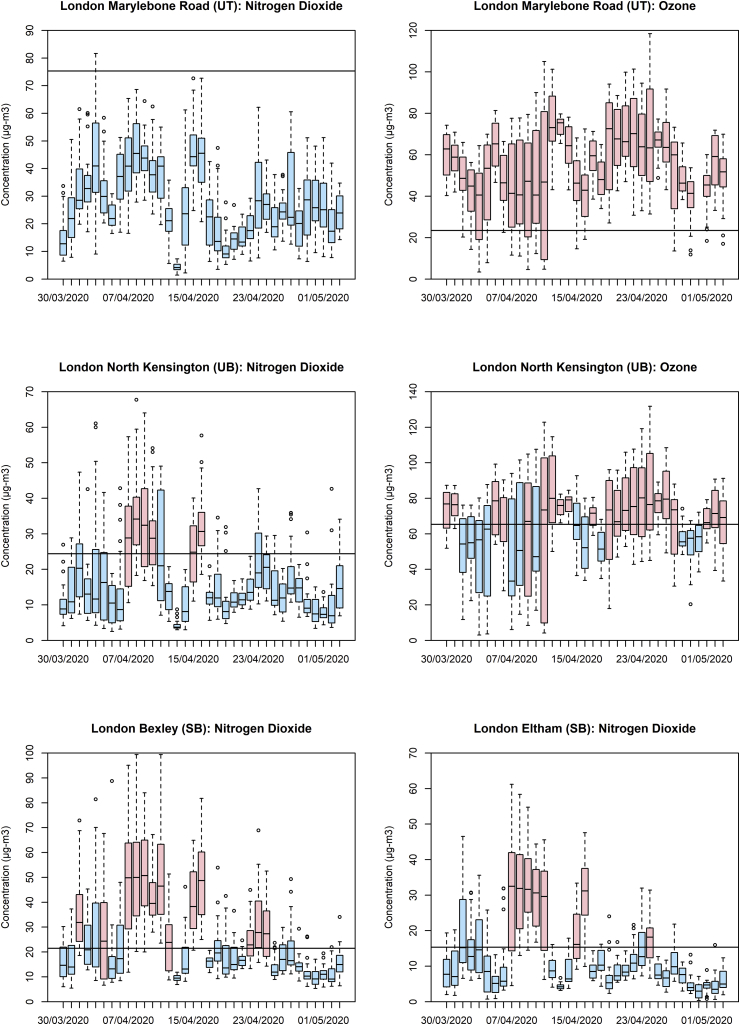
Box-and-whisker plots comparing the distribution of hourly pollution concentrations for days in weeks 14–18 of 2020, with the median daily-median concentration for a comparable period in 2017–19.

Greater London is the only built-up-area to report measurements across the full range of urban settings, with sites that are within 30 km of each other (instead of in different and potentially unconnected cities). Fig. 1 presents a series of box-and-whisker plots for monitoring sites in London, comparing the distribution of hourly NO_2_ and O_3_ concentrations for days in weeks 14–18 of 2020, with the median daily-median concentration for a comparable period in 2017–19. The boxes represent the interquartile range of the hourly measurements (excluding 25% of the highest and 25% of the lowest values), and indicate the variability on any given day. Daily-median values of NO_2_ at London Marylebone were consistently below the expected value of 75 μg/m^3^, with the reverse observed for O_3_ as daily-medians exceed the threshold of 23 μg/m^3^. The whiskers indicate that 95% of the hourly NO_2_ measurements per day are below the expected threshold, except for those on 03/04/2020. The urban background site at North Kensington shows a general reduction on levels of NO_2_ reported in previous years, with the exception of a four (07/04/2020 to 10/04/2020) and two consecutive day (15/04/2020 to 16/04/2020) period exceeding the daily-median threshold of 24 μg/m^3^. Levels of O_3_ are generally higher than expected outside of these two periods, and a few days at the start of April. These periods of elevated NO_2_ concentrations are more apparent at suburban background sites, with a third spike reported from the 22/04/2020 to 24/04/2020 observed at London Bexley. Although daily levels have generally declined, the contrast is not as stark as urban traffic sites. A number of hourly observations remain consistently high for most days at these background sites, indicative of essential or local short-distance trips.

Limited measurement information on VOCs, such as the BTEX species (benzene, toluene, ethylbenzene and o-xylene), were available from three of the four active sites in the UK Automatic Hydrocarbon Network. BTEX measurements were generally lower than expected for this time of the year in London (see Table S9). However, days coinciding with the three NO_2_ episodes, equalled or exceeded the daily-average BTEX concentrations of previous years. VOCs are known to promote photochemical reaction mechanisms, and in part perhaps explain why several days of elevated NO_2_ concentrations were observed.

Despite the overall improvements in NO_2_ and PM_2.5_, at urban background sites 15.9% and 25.8% of the valid measurement days over the 5-week intervention in 2020, respectively, reported equal to or higher than expected concentrations of NO_2_ and PM_2.5_ reflecting the importance of meteorology. A slightly larger number of consecutive days with high pollutant concentrations were recorded at suburban sites (see Table 1), where meteorological influences were further confounded by increasingly localised population movements. As previously noted, episodes involving NO_2_ corresponded with improvements in O_3_.

### 3.2 Business-as-usual scenario models

The effect of the travel restrictions on pollutant concentrations is considered with respect to a business-as-usual scenario (BAU), where local emission sources continue to operate as normal under the measured meteorological conditions.

Site specific BAU models were developed using the long-term trend, seasonal, weekday and hourly temporal structures, in conjunction with the aforementioned meteorological and pollutant measurements recorded from 01/01/2017 to 31/12/2019. Table S10 shows the average relative influence of each of these model parameters on UK pollution concentrations. Reoccurring temporal structures appear to explain 41–47% of the variation in nitrogen oxides and fine particulate concentrations, with meteorological factors explaining the remaining 53–58%. Wind speed, wind direction and temperature are the main meteorological drivers, with favourable conditions reported throughout weeks 15–17 in England and Wales (see Table S6). O_3_ concentrations are highly influenced by meteorological events, which appear to explain up to 69% of the variation in hourly concentrations. The models report relative humidity as the main meteorological driver of O_3_, which is a function of temperature and pressure.

Table 2 summarises the average daily-difference between measured concentrations and the forecast estimates, by site type. Day-to-day comparisons at urban background sites identified similar magnitudes of NO_2_ reductions and O_3_ gains reported by the comparison of measurement data, which were respectively estimated across all sites at −35.7% (95% CI: −44.9% to −22.8%) and +8.3% (95% CI: −1.2 to +21.1). For reference, NO_2_ and O_3_ concentrations measured over lockdown, respectively reported a −36.7% and +7.4% change on the mean daily-average concentration measured across comparable periods in the three previous years. Day-to-day contrasts at urban traffic sites were associated with average NO_2_ reductions of −38.4% (95% CI: −47.0% to −16.9%), and O_3_ gains of +22.4% (95% CI: +10.0% to +38.1%). These are somewhat smaller than the NO_2_ reductions of −47.9% and O_3_ gains of +34.1% recorded by the comparison of measurement data, albeit within the 95% confidence intervals of the BAU forecasts. Of the three O_3_ urban traffic measurement sites, substantial gains were estimated at London Marylebone road (95% CI: +27.1% to +53.9%) and Birmingham A4540 Roadside (95% CI: +12.7% to +49.8%), but not for Exeter roadside (95% CI: −9.8% to +10.5%). Larger differences were reported in relation to the reduction of fine particulate matter, with the BAU models estimating PM_2.5_ to reduce at all sites by −42.9% (95% CI: −47.9% to −37.0%), compared to the −16.5% reduction identified by the comparison of measurement data.

**Table 2:** Comparing measured with modelled air pollution concentrations under a ‘business-as-usual’ (BAU) scenario for weeks 14–18 in 2020, summarised by site type across England, Scotland, Wales, and Northern Ireland.

Site Type	5-week average of the daily estimates, under a ‘business-as-usual’ scenario in μg/m^3^ (AURN count)				% change on ‘business-as-usual’ daily concentration estimates				Absolute change on ‘business-as-usual’ daily concentration estimates (μg/m^3^)				% days below the ‘business-as-usual’ daily concentration estimates			
	NO_2_	NO_X_	O_3_	PM_2.5_	NO_2_	NO_X_	O_3_	PM_2.5_	NO_2_	NO_X_	O_3_	PM_2.5_	NO_2_	NO_X_	O_3_	PM_2.5_
Rural Background	11.6 (5)	10.5 (5)	69.3 (16)	26.6 (3)	−43.5	−36.7	+12.0	−47.8	−8.9	−6.1	+7.4	−24.4	92.0	87.4	41.4	89.5
Suburban Background	22.8 (2)	27.6 (1)	63.8 (2)	34.4 (2)	−20.4	+31.1	+8.1	−57.8	−5.8	+6.5	+4.8	−47.1	65.7	62.9	38.6	98.6
Urban Background	20.9 (26)	33.6 (16)	64.9 (30)	21.0 (17)	−35.7	−36.4	+8.3	−40.7	−11.6	−19.2	+5.0	−14.4	89.0	90.9	45.0	88.9
Urban Traffic	32.6 (38)	88.3 (30)	49.7 (3)	21.1 (5)	−38.4	−51.0	+22.4	−41.5	−20.3	−91.9	+9.1	−15.0	93.3	95.0	22.9	89.1
Suburban Industrial	18.9 (1)	–	73.7 (1)	–	−37.6	–	−13.5	–	−11.4	–	−11.5	–	91.4	–	88.6	–
Urban Industrial	11.9 (1)	8.1 (1)	69.2 (3)	–	−2.0	+67.2	−0.1	–	−0.2	+3.3	−0.1	–	52.9	68.6	47.6	–
All Sites	26.2 (73)	61.8 (53)	65.7 (55)	22.6 (27)	−36.8	−41.4	+9.3	−42.9	−15.3	−43.7	+5.6	−17.0	90.3	92.0	43.4	89.7

**Fig. 2:**
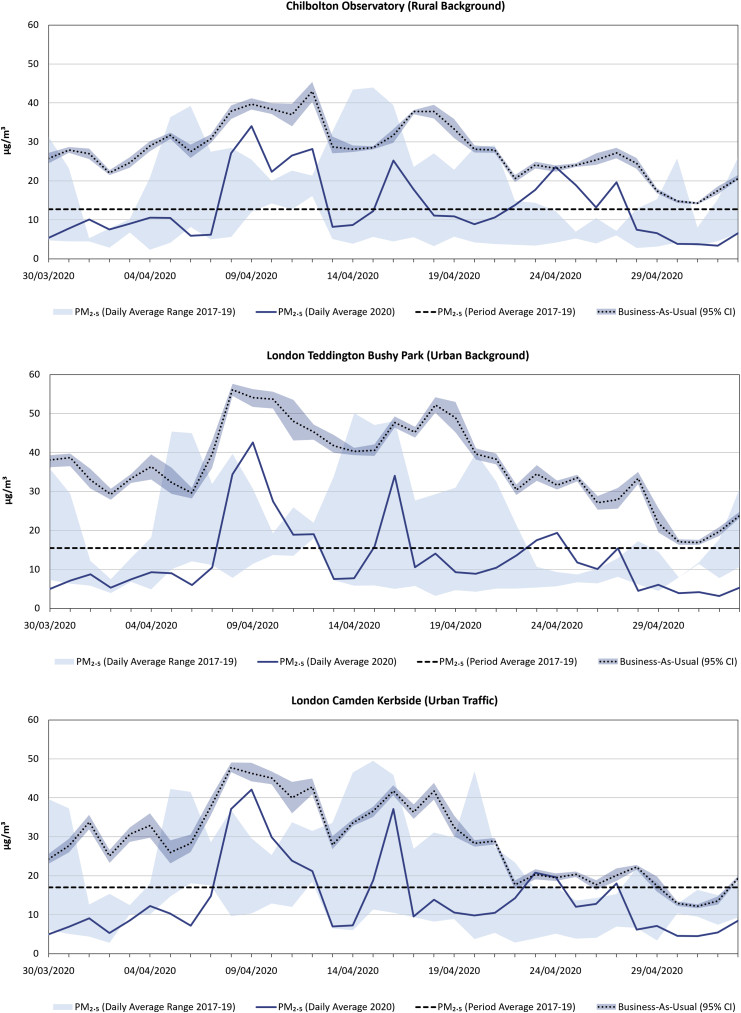
Time-series plots of measured daily-average PM_2.5_ concentrations for weeks 14–18 of 2020, in relation to the range of measurements for corresponding weekdays in 2017–19, and estimates under a business-as-usual (BUA) scenario.

**Fig. 3:**
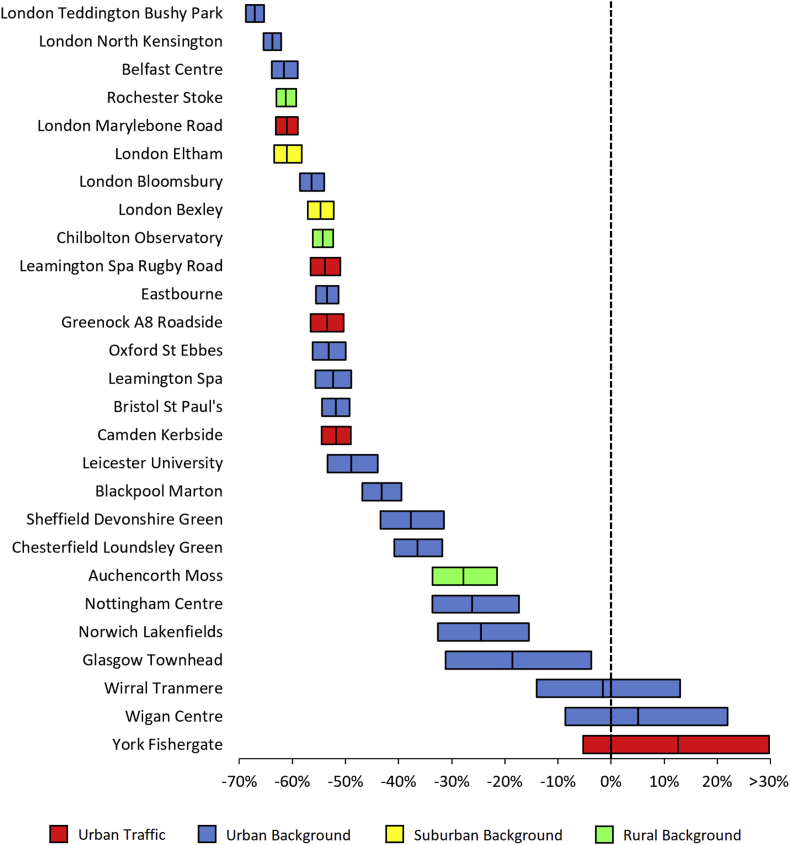
Average daily percentage change of measured PM_2.5_ concentrations on forecasted values under a business-as-usual (BUA) scenario with 95% confidence intervals, considering the observed meteorological conditions.

Fig. 2 presents time-series plots of daily-average PM_2.5_ measurements across weeks 14–18 of 2020, in relation to the (a) range of concentration measurements for corresponding weekdays in 2017–19, and (b) estimates under a BAU scenario. Like Fig. 1, three short periods of consecutively high measurement days are identified, from 08/04/2020 to 12/04/2020 (week 15), 15/04/2020 to 17/04/2020 (week 16), and 23/04/2020 to 24/04/2020 (week 17). These coincide with the meteorologically stagnation of local air, and the importing of pollution in continental air masses. The time-series plots show that meteorological events such as this are commonplace for this time of year, and that PM_2.5_ pollution events will occur even with successful policy driven reductions in road-transport. Fig. 3 shows the average daily percentage change of measured PM_2.5_ concentrations on the BAU estimates with 95% confidence intervals, considering the observed meteorological conditions. Reductions are observed across all sites with statistically stable estimates, the magnitude of which is independent of region or site type.

## 4 Discussion

In this analysis, we examined how air quality has changed across the UK in relation to travel restrictions, imposed to slow the spread of the SARS-CoV-2 virus. The 5-week period of the most stringent restrictions saw an overall improvement in air quality for NO_2_ and PM_2.5,_ but not for O_3_, when compared to the reference period (i.e. the same 5 weeks in 2017–2019). Similar magnitudes of change were observed when comparing measured NO_2_ and O_3_ concentrations to their modelled estimates, under a business-as-usual (BAU) scenario. Daily PM_2.5_ concentrations were consistently lower than their BAU estimates, but this reduction was 2.6 times greater than the comparisons using reference data. This highlights the importance of accounting for local and regional meteorological conditions, alongside any direct comparison of measurements from previous years.

### 4.1 Changes in air quality during lockdown

Over the 5-week period, improvements in air quality were largest at urban traffic sites for NO_2_ and more modest at other locations (more representative of where most of the population live). At a few sites air quality worsened but overall there were more modest improvements in concentrations of PM_2.5_. O_3_ was found to increase at urban background and traffic sites. NO_x_ emissions from road traffic are mainly NO, with a smaller proportion being emitted as NO_2_. Emissions of NO react with and deplete O_3_, usually resulting in generally lower levels of O_3_ at roadside than at background sites. Reductions in NO_x_ emissions from road traffic during the 5-week period led to increases in O_3_ at roadside sites because less O_3_ is used up in reactions with NO (AQEG, 2009), although this observation is limited to 3 roadside sites as the focus of monitoring in the AURN for O_3_ is urban background and rural locations. It is difficult to gain more insight on why levels of O_3_ concentrations were also elevated at background sites, especially in Southern and Eastern England, without undertaking a detailed analysis of O_3_ combined with sunshine duration/intensity, wind speed/direction, and VOCs. This is hampered because VOCs are not routinely measured alongside other pollutants.

There were also periods of elevated concentrations of PM_2.5_ and O_3_ during the lockdown. These periods are often referred to as air pollution ‘episodes’ usually last several days and occasionally more than a week. They occur in warm and cold seasons as air stagnates under high pressure (i.e. anticyclones) and are common in spring and summer in the UK. Locally, O_3_ is formed by photochemical reactions between NOx emissions and VOCs driven by sunny conditions. Easterly winds, can also draw polluted air from Central and Eastern Europe leading to elevated levels of PM_2.5_ and O_3_, especially in Southern England (Pope et al., 2016; Graham et al., 2020). Elevated concentrations around Easter 2020 (April 08, 2020 to April 12, 2020, and April 15, 2020 to April 17, 2020) were associated with easterly air flow as shown in meteorological data from stations in the South East of England. Although there was a general decrease in measured PM_2.5_ concentrations, our data showed a number of consecutive days above the expected daily average based on previous years.

The effect of the ‘lockdown’ on pollutant concentrations was considered with respect to a BAU scenario, where the contributions of normal anthropogenic activities were estimated given the local meteorological conditions observed in weeks 14–18 of 2020. The BAU scenario estimates showed that the meteorological conditions in April 2020 had a higher than average influence on the formation of these air pollution episodes, but concentrations seen were still within the upper threshold of prior years.

Stedman (2003) and Fenech et al. (2019) have previously documented the importance of meteorological drivers on warm weather pollution episodes in the UK, which our study implies will continue to prevail even if reductions to vehicle fleet emissions are achieved. Fenech et al. (2019) reported a 36–56% increase in short-term exposures to concentrations of O_3_ and PM_2.5_, compared to data from previous years, during UK pollution episodes under anticyclonic conditions on 01/07/2006 to 05/07/2006 and 18/07/2006 to 22/07/2006. They estimated that this short-term exposure to PM_2.5_ and O_3_ corresponded with 3.4–5.2% and 1.6–3.9% increases in all-cause mortality, respectively. Meanwhile, Stedman (2003) estimated that poor air quality was accountable for 21–28% of the total excess deaths in England and Wales during a two-week heatwave in August 2003 (423–769 deaths), where population-weighted mean O_3_ and PM_2.5_ concentrations reached 103 μg/m^3^ (+77%) and 29 μg/m^3^ (+81%), respectively. The implications of this are:

(a) Weather conditions may contribute to air pollution episodes even if future emission reduction targets from the road-transport and other sectors are achieved;
(b) Weather effects need to be carefully considered by policy makers when quantifying improvements to air quality estimated from modelled scenarios, especially as global temperatures continue to rise;
(c) Given the contribution of continental air masses to air quality in the UK, especially for PM_2.5_ and O_3_, there is a need for continued cooperation on international and European air quality improvement initiatives.

Despite these episodes, there was an overall improvement for NO_2_ and PM_2.5_ attributed to the curtailing of non-essential travel, following governmental advice to stay at home. As such, the largest decreases in pollutant concentrations were observed for inner-city locations and other places of commerce, as short local trips became the new norm.

Reductions in industrial activity will also have contributed to air quality changes. Measurement data from the two active suburban industrial sites, located in Horley (Surrey) and Sharston (Greater Manchester), also reported reductions in nitrogen-based pollutants that were comparable to urban traffic sites. These are perhaps indicative of reductions in on-site operations and traffic volume from a reduced workforce, further reflecting the changes to travel. Although it is difficult provide more insight on changes in concentrations related to industrial activity they appear in line with national productivity reports (ONS, 2020; Eurostat, 2020).

Based on BAU modelling, day-to-day comparisons across all site types reported reductions in NO_2_ of −36.8% (95% CI: −45.5 to −19.8) and gains in O_3_ of +9.3% (95% CI: +0.1 to +21.6), which are comparable to the differences in the measurement data (see Table 1, Table 2). In contrast, the BAU scenarios predicted that particulate concentrations would have normally been a lot higher if the lockdown travel restrictions had not occurred, given the anticyclone weather systems; as indicated by the observed PM_2.5_ concentrations being 42.9% lower than the BAU model estimates (95% CI: −47.9% to −37.0%), and only 16.5% lower than measurements in previous years (see Table 1, Table 2). Although April 2020 was unusually warm and sunny, such weather systems are common for this time of year, and are expected to drive particulate events even if there are reductions in road-transport emissions.

Another consideration is the impact of urban vegetation, which can influence O_3_ concentrations through the process of dry deposition (sink), or the release of precursor compounds (source). Air quality datasets frequently identify a coincidence of high surface ozone and low relative humidity – under these meteorological conditions trees close their stomata to prevent drying out, limiting the removal of O_3_ from dry deposition (Kavassalis and Murphy, 2017). Meanwhile, O_3_ precursors in the form of biogenic volatile organic compounds (BVOC), are released during warm seasons, flowering periods, and in response to environmental stressors such as drought and insect outbreaks (Fitzky et al., 2019). It was estimated that BVOCs contribute to 17–20% of the daily maximum 8-h O_3_ concentrations in Berlin under typical summertime temperatures of 22–24 °C, and can be responsible for up to 60% of the O_3_ quota during heatwaves (Churkina et al., 2017). Although future legislation may reduce levels of anthropogenic VOCs, there is the potential for these O_3_ precursors to be offset by the contributions from biogenic sources as global temperatures rise. It was advised that urban tree planting schemes in the UK carefully select species based on their BVOC emission and NO_X_ scavenging potential in the urban atmosphere (Owen et al., 2003).

### 4.2 Strengths and limitations

A major strength of our study is that it provides a national comparison of changes in air quality during lockdown with respect to: (a) measurements from the three previous years, and (b) modelled concentrations from a BAU scenario given the weather patterns during lockdown. Limitations apply to BAU, a model with its own uncertainty. As a minimum, interpreted BAU models were able to account for 75% of the variation in the estimates of hourly pollutant concentrations under a series of specified temporal and meteorological parameters. The uncertainty in these hourly estimates is shrunk when converting to a daily mean, using a bootstrapped approach. The 95% confidence intervals for NO_2_, NO_x_, O_3_ and PM_2.5_ at each site, on average differ from the mean-daily estimate by 15.7%, 24.3%, 9.7% and 8.4%. Any comparisons between the daily measurement data and BAU estimates, take these 95% confidence intervals into account. We suggest that our results are interpreted as indicative of what air quality would have been like during weeks 14–18 of 2020 without a COVID-19 related lockdown. The forecasting of atmospheric pollutant concentrations is a non-trivial procedure involving many interacting nonlinear stochastic processes, which are further complicated by chemical transformations. BRT models are however capable of modelling such complex multivariable interactions, and are now widely implemented air pollution forecasts (Carslaw and Taylor, 2009; Carslaw et al., 2012; Ibrahim et al., 2019).

A further strength of this study is that it used monitoring station measurements with information on site types. Some other studies have used satellite data to document changes in air quality on a regional/global scale (Muhammad et al., 2020; Venter et al., 2020) but due to their relatively coarse spatial resolution they are unable to distinguish changes by site type (e.g. roadside versus background) which we were able to for the UK.

While we were able to compare changes regionally in air quality and with traffic reported for major roads (i.e. ‘A’ roads and motorways), a limitation is that we did not have information on secondary and minor roads. We may therefore have underestimated the variability across the road network in different regions when comparing changes in air quality with changes in road traffic. Furthermore, we did not have detailed data on changes in domestic and industrial emissions so were unable to explore spatial contrasts (i.e. by site type and by region) in concentration changes for these sources. This has limited our capacity to interpret changes in urban background locations, especially for O_3_ as we lack data on measured VOCs.

### 4.3 Comparison with other studies

A call for evidence from UK Government on changes in air quality during lockdown received contributions from 50 organisations, using a mixture of measurements from the AURN, modelling and satellite data, and was summarised by the UK Air Quality Expert Group (AQEG) in June 2020 (AQEG, 2020). Reductions in NO_2_ were reported to be 20–30% with more moderate reductions reported for PM_2.5_ of 2–5 μg/m^3^ in Southern England than would have been expected under a BAU scenario. The improvements for NO_2_ given in the AQEG report are more modest than from our analysis, which uses a different modelling framework: AQEG removed weather effects from their pollution datasets with ‘detrending’ models, whereas our analysis created a direct comparison period by including the observed meteorological conditions in a business-as-usual pollution model. Increased levels of O_3_ were also noted at some locations based on modelling.

Although our analysis shows general improvements in particulate pollution across the UK, a recent analysis of monitoring stations operated by Scottish local authorities reported daily-average PM_2.5_ concentrations that were comparable to previous years (6.6 μg/m^3^) (Dobson and Semple 2020). These findings are indicative of localised meteorological differences between southern England and Scotland.

Several other studies have documented the impact of COVID-19 lockdowns on air quality in Brazil, India and Spain. Baldasano (2020) reports that NO_2_ concentrations at measurement sites respectively fell by 50% and 62% in Barcelona (9 sites) and Madrid (24 sites), under Spanish COVID-19 lockdown measures during March 2020. These values coincide with a 75% and 77% reduction in main road traffic levels across Barcelona and Madrid, respectively. Sharma et al. (2020) analysed the effect of lockdown across 22 cities in India, reporting reductions on the regional air quality index (AQI) of between 15% and 44%. Dantas et al. (2020) analysis of the first week of lockdown in the Brazilian city of Rio de Janeiro, respectively reported an average reduction of 24% and 43% on the daily-median PM_10_ and NO_2_ concentrations at 3 monitoring stations (<100 m from a major road), with O_3_ rising by 18%, compared to measurement data for the first week of March 2020. Further analysis of the data is restricted, as compliance fell in later weeks following a change in government messages. The analysis also fails to consider the influence of metrological parameters, which restricted the transport of pollutants from nearby industrial areas, resulting in larger reductions. Muhammad et al.‘s (2020) comparison of Sentinel-5P satellite measurement data in March 2019 and 2020, implies that NO_2_ emissions reduced in France, Spain and Italy by 20–30%, in accordance with an 82–89% reductions in travel across the national road-networks, but there is no commentary on how these reductions to reductions in ground-level concentration. Despite any shortcoming, these studies report different levels of changes in air quality but the trend is similar to our findings for the UK.

### 4.4 Interventions to reduce emissions and policy implications

In an effort to comply with air quality limit values, and to protect human health, a number of European cities have in recent years introduced planned traffic interventions in the form of low emission zones (LEZs). These restrict the entry of vehicles based on the emission standard the vehicles were originally constructed to meet, using active (number plate recognition) or passive (permit) enforcement measures. These measures tend to be applied at city-region scale. Examples include the London Congestion Charge Zone (CCZ) which applied only to the centre of London and the later London-wide LEZ, LEZs in the German cities of Berlin, Cologne and Hannover, emission abatement systems for heavy duty vehicles in Denmark and the Netherlands, and removal of older heavy duty vehicles in Scandinavian cities such as Stockholm.

The London CCZ yielded a 15% decrease in vehicle km two years after its implementation, resulting in a 12% reduction in NOx emissions; however emissions were observed to increase on the inner ring road by 1.5%, as some traffic is redistributed across the network (Beevers and Carslaw, 2005). It was estimated that street-level NO_2_ concentrations in the Danish cities of Aalborg, Aarhus, Copenhagen, and Odense reduced by 4–11%, through the enforcement of enforcing Euro-III standards on heavy duty vehicles (Jensen et al., 2011). LEZ’s targeting < EURO-IV trucks across the Netherlands, on average reported a 9.8% reduction in traffic intensities resulting in a 6.2% reduction in NO_2_ concentrations, two-years after their implementation (Boogaard et al., 2012). NO_2_ and PM_2.5_ concentrations were respectively found to reduce in the Stockholm LEZ by 4% and 26% over the period 1995–2001 (Rapaport, 2002). LEZs were implemented in the German cities of Berlin, Cologne and Hanover in 2008, in response to revised EU directives on ambient air quality and fine particles. While the LEZs has had no measurable impact on traffic flows, the turnover of the national fleet towards cleaner vehicles has speeded up considerably. In terms of local environmental benefits, areas within the Berlin ‘Passive’ LEZ have been estimated to experience a 4.5% reduction in PM_10_ (Lutz and Rauterberg-Wulff, 2010).

The improvements for air quality seen in LEZs are relatively modest compared to those reported from our study, implying that much larger interventions are needed that are scalable nationally and internationally to achieve the magnitude of overall improvement in air quality seen during lockdown. The challenge for reducing air pollution, especially for PM_2.5_ and O_3_, is international and major interventions are likely to result in modest improvement in air quality.

Major improvements to air quality may come from switching to a ‘greener’ vehicle fleet. Road traffic emission reductions during lockdown may indicate what could be achieved for air quality by having a high proportion (e.g. >50%) of the vehicle fleet switching to electric. Benefits overall to air quality are however likely to be lower than seen during lockdown as BAU traffic volumes would result in normal levels of congestion resulting in higher tailpipe emissions for non-electric vehicles associated with lower traffic speeds and poorer ventilation around roads (AQEG 2019; Grigoratos and Martini 2014). However, the switch to an electric fleet is not without its own problems. Although for electric vehicles there are no tail-pipe emissions, substantial particulate contributions still arise from the tyres, brakes and general wear and tear of any vehicle (Soret et al., 2014; Timmers and Achten, 2016). The percentage of fleet electrification needed to acquire a significant level of improvement is between 26 and 40%, highlighting the difficulties in developing sustainable interventions for improving air quality (Soret et al., 2014). There are also issues relating to how this energy is sourced, especially when fossil fuels still account for 51% of electricity produced in the UK (BEIS, 2017), for example.

## 5 Conclusions

With respect to implications of findings for future transport policy initiatives, air quality changes seen during the lockdown period, that in the UK primarily affected transportation, reinforce the need to ensure actions across all emissions sectors for sustained substantive air quality improvements. Given the contribution of long-range transport of continental air masses to air quality in the UK especially for PM_2.5_ and O_3_, close cooperation with international and European air quality improvement initiatives is needed. Weather conditions may contribute to particulate episodes even at much reduced levels of vehicle emissions and need to be considered in scenarios modelling future air quality in differing emission reduction policies. Routine VOC monitoring would help in monitoring and predicting changes in O_3_ in relation to emission controls.

## Funding statement

This study is part-funded by the 10.13039/501100000272National Institute for Health Research (NIHR) Health Protection Research Unit in Environmental Exposures and Health, a partnership between 10.13039/501100002141Public Health England, the 10.13039/501100000869Health and Safety Executive and the 10.13039/501100000738University of Leicester.

## Credit author statement

Calvin Jephcote: Conceptualization, data curation, methodology, software, formal analysis, validation, writing (original draft, review and editing), visualization, Anna L Hansell: Conceptualization, writing (review and editing), supervision, funding acquisition, Kathryn Adams: visualization, writing (original draft), resources, investigation. John Gulliver: Conceptualization, methodology, validation, writing (original draft, review and editing), visualization, supervision, funding acquisition

## Declaration of competing interest

The authors declare that they have no known competing financial interests or personal relationships that could have appeared to influence the work reported in this paper.
