# Supplementary material for: Changes in air quality during COVID-19 ‘lockdown’ in the United Kingdom
Source: Environ Pollut. 2021 Mar 1;272:116011. doi: 10.1016/j.envpol.2020.116011 (PMC7677678; doi:10.1016/j.envpol.2020.116011)
Supplement: Multimedia component 1 [file mmc1.docx]

**Supplementary materials: Changes in air quality during COVID-19 ‘lockdown’ in the United Kingdom**

Calvin Jephcote ^[1]^, Anna Hansell ^[1, 2]^, Kathryn Adams ^[1]^, John Gulliver ^[1, 2]^

1. Centre for Environmental Health and Sustainability, University of Leicester, United Kingdom
2. NIHR HPRU Centre for Environmental Exposures and Health, University of Leicester, United Kingdom

| **Contents** |  | **Description** |  | **Page** |
| --- | --- | --- | --- | --- |
| Table S1 |  | Cross-tabulation of automatic air pollution analysers with 95% capture rates, by site type and pollutant measurement data, in relation to the number of active UK-AURN sites for each category (%). |  | S2 |
| Table S2 |  | UK Automatic Urban and Rural Network (AURN) site classification system. |  | S3 |
| Table S3 |  | Boosted Regression Tree (BRT) model development. |  | S4 |
| Table S4 |  | Boosted regression tree model performance summaries for the estimation of hourly pollutant concentrations in 2017-19. Cross-validation measures include: factor-of-two (F2), coefficient of efficiency (COE), index of agreement (IOA), and r-squared (R^2^ ≥ 0.75). |  | S5, S6 |
| Table S5 |  | Changes in monthly average daily traffic (ADT) counts on A-Roads and Motorways, as reported by Highways England via WebTRIS. |  | S7 |
| Table S6 |  | Selection of weekly-average meteorological profiles at urban background sites across the UK, for weeks 14 to 18 of 2020. Wind direction is calculated as a scalar average. |  | S8 |
| Table S7 |  | Regional summary of urban background sites in England, Scotland, Wales, and Northern Ireland. |  | S9 |
| Table S8 |  | Regional summary of urban traffic sites in England, Scotland, Wales, and Northern Ireland |  | S10 |
| Table S9 |  | UK Automatic Hydrocarbon Network provisionally verified summaries of benzene (B), toluene (T), ethylbenzene (E), and o-xylene (X). |  | S11 |
| Table S10 |  | The average relative influence of each variable on UK pollution concentrations, as reported by boosted regression tree (BRT) models of AURN sites with a cross-validation r-squared ≥ 0.75. |  | S12 |
| Figure S1 |  | Map of the United Kingdom detailing the Government Office Regions (GOR), excluding Northern Ireland. |  | S13 |
| Figure S2 |  | Regional summary of percentage changes in NO_2_ and PM_2.5_ concentration across traffic and background AURN sites. |  | S14 |

**Table S1:** Cross-tabulation of automatic air pollution analysers with 95% capture rates, by site type and pollutant measurement data, in relation to the number of active UK-AURN sites for each category (%).

| Site Type | NO_2_ | NO_x_ | O_3_ | PM_2.5_ | All Pollutants |
| --- | --- | --- | --- | --- | --- |
| Rural Background | 9 (64%) | 11 (79%) | 16 (80%) | 3 (60%) | 19 (95%) |
| Suburban Background | 3 (75%) | 3 (75%) | 2 (67%) | 2 (100%) | 4 (100%) |
| Urban Background | 41 (65%) | 41 (65%) | 31 (72%) | 28 (64%) | 47 (72%) |
| Urban Traffic | 47 (70%) | 47 (70%) | 3 (100%) | 11 (46%) | 51 (73%) |
| Suburban Industrial | 2 (100%) | 2 (100%) | 1 (100%) | - | 2 (100%) |
| Urban Industrial | 5 (56%) | 6 (67%) | 3 (75%) | 3 (50%) | 6 (67%) |
| All Sites | 107 (67%) | 110 (69%) | 56 (76%) | 47 (58%) | 129 (76%) |

**Table S2:** UK Automatic Urban and Rural Network (AURN) site classification system.

| The Department for Environment, Food and Rural Affairs use the following three-fold classification for monitoring stations in the UK-AURN:   - Urban areas comprised of buildings with at least two floors on either side (i.e. street canyons), representative of air quality within a few km^2^. Urban sites measuring ozone may include open areas, located away from established vegetation. - Suburban areas are contiguous settlement of detached buildings of any size mixed with non-urbanised areas (i.e. agriculture, lakes, and woodland), representative of air quality within tens of km^2^. - Rural areas target the protection of natural ecosystems and must be sited >5 km away from built-up areas, industrial installations, motorways or major roads. They represent air quality within a surrounding area of at least 1,000 km^2^.   These environments may be further separated into traffic, industrial and background sites. These types of environment are either indicative of a predominant source of pollution in the area, or considered to represent background levels if the site is typically upwind of such pollutant sources.  There are six types of measurement site in the UK-AURN: urban traffic (UT), urban industrial (UI), urban background (UB), suburban-industrial (SI), suburban background (SB), rural background (RB).  Additional information on the environment types used for classifying air quality monitoring sites can be found at: <https://uk-air.defra.gov.uk/networks/site-types> |
| --- |

**Table S3:** Boosted Regression Tree (BRT) model development.

| The forecasting of atmospheric pollutant concentrations is a non-trivial procedure involving many interacting nonlinear stochastic processes, which are further complicated by chemical transformations. Machine learning methods, in the form of Boosted Regression Trees (BRT), are capable of modelling such complex multivariable interactions, and are now widely implemented air pollution forecasts (Carslaw et al 2009, Carslaw et al 2012, Ibrahim et al 2019).  BRT models combine the strengths of two techniques: decision tree algorithms that relate a response to their predictors by recursive binary splits, supplemented with boosting methods to improve predictive performance (Elith et al 2008). The boosting approach carries forward the error in prediction of the previous decision tree, to facilitate iterative improvements in model accuracy. Unlike many machine learning techniques, the BRT approach is completely transparent with the interactions between variables determined, ranked and visualised. BRT models were constructed in R-Statistical Software, using the ‘gbm’ v2.1.8 package.  In accordance with literature guidelines, suitable BRT models may be constructed from 1,000 decision trees, with an interaction depth of 6, and a shrinkage value of 0.1 (Friedman 1999, Hastie et al 2001, Elith et al 2008). These thresholds were used to develop exploratory BRT models, predicting hourly concentrations of NO_2_, O_3_, and PM_2.5_ at the previously analysed UK-AURN monitoring sites under a ‘Business-as-usual’ (BAU) scenario. This scenario was developed from a long-term trend, seasonal, weekday and hourly temporal structures, in conjunction with the aforementioned meteorological and pollutant measurements recorded across the period of 01/01/2017 to 31/12/2019. 75% of the hourly observations were used to train the BRT models, with 25% of the data used to cross-validate the machine learning approach. The final models were calibrated using the hourly measurements from 2017 to 2019.  Air quality forecasts for the period of 30/03/2020 to 03/05/2020 are considered to represent the influence of local source contributions under normal levels of human activity, given the observed meteorological conditions. BAU daily mean concentrations were obtained from a boot-strapping approach of the hourly predicted estimates, using 10,000 iterations. These BAU daily estimates were then compared to the measured daily concentrations. Uncertainties in the predictions and model performance were considered to determine whether the short-term travel intervention can reliably be quantified in terms of its effects on local air pollution. Only, models with a cross-validation R^2^ ≥ 0.75 were deemed suitable for forecasting at any given location. |
| --- |

**Table S4:** Boosted regression tree model performance summaries for the estimation of hourly pollutant concentrations in 2017-19. Cross-validation measures include: factor-of-two (F2), coefficient of efficiency (COE), index of agreement (IOA), and r-squared (R^2^ ≥ 0.75).

| SITE | Nitrogen Dioxide (NO_2_) | | | | Nitrogen Oxides (NOx) | | | | Ozone (O_3_) | | | | Fine Particulates (PM_2.5_) | | | |
| --- | --- | --- | --- | --- | --- | --- | --- | --- | --- | --- | --- | --- | --- | --- | --- | --- |
|  | F2 | COE | IOA | R^2^ | F2 | COE | IOA | R^2^ | F2 | COE | IOA | R^2^ | F2 | COE | IOA | R^2^ |
| ABD7 | 0.94 | 0.56 | 0.78 | 0.77 | 0.86 | 0.57 | 0.78 | 0.77 |  |  |  |  |  |  |  |  |
| ABD8 | 0.9 | 0.6 | 0.8 | 0.8 | 0.8 | 0.59 | 0.8 | 0.78 |  |  |  |  |  |  |  |  |
| ACTH |  |  |  |  |  |  |  |  | 1 | 0.58 | 0.79 | 0.81 | 0.78 | 0.49 | 0.75 | 0.77 |
| AGRN | 0.91 | 0.58 | 0.79 | 0.79 | 0.89 | 0.54 | 0.77 | 0.77 | 0.92 | 0.59 | 0.8 | 0.83 |  |  |  |  |
| AH | 0.86 | 0.63 | 0.82 | 0.85 | 0.91 | 0.64 | 0.82 | 0.85 | 1 | 0.59 | 0.79 | 0.83 |  |  |  |  |
| ARM6 | 0.92 | 0.57 | 0.79 | 0.8 | 0.85 | 0.56 | 0.78 | 0.77 |  |  |  |  |  |  |  |  |
| BAR3 | 0.92 | 0.54 | 0.77 | 0.76 |  |  |  |  | 0.92 | 0.59 | 0.79 | 0.82 |  |  |  |  |
| BBRD | 0.88 | 0.59 | 0.8 | 0.8 | 0.84 | 0.56 | 0.78 | 0.77 |  |  |  |  |  |  |  |  |
| BDMA | 0.96 | 0.6 | 0.8 | 0.81 | 0.92 | 0.59 | 0.79 | 0.78 |  |  |  |  |  |  |  |  |
| BEL1 | 0.93 | 0.6 | 0.8 | 0.81 | 0.83 | 0.58 | 0.79 | 0.78 |  |  |  |  |  |  |  |  |
| BEL2 |  |  |  |  |  |  |  |  |  |  |  |  | 0.88 | 0.49 | 0.75 | 0.75 |
| BEX | 0.91 | 0.57 | 0.79 | 0.78 | 0.85 | 0.53 | 0.76 | 0.77 |  |  |  |  | 0.9 | 0.56 | 0.78 | 0.8 |
| BIRR |  |  |  |  |  |  |  |  | 0.85 | 0.62 | 0.81 | 0.84 |  |  |  |  |
| BLC2 | 0.9 | 0.56 | 0.78 | 0.77 | 0.86 | 0.52 | 0.76 | 0.76 | 0.95 | 0.59 | 0.79 | 0.82 | 0.8 | 0.47 | 0.74 | 0.76 |
| BORN | 0.86 | 0.56 | 0.78 | 0.78 |  |  |  |  | 0.93 | 0.6 | 0.8 | 0.82 |  |  |  |  |
| BR11 | 0.95 | 0.58 | 0.79 | 0.79 |  |  |  |  |  |  |  |  |  |  |  |  |
| BRS8 | 0.94 | 0.54 | 0.77 | 0.75 |  |  |  |  | 0.89 | 0.58 | 0.79 | 0.8 | 0.88 | 0.51 | 0.75 | 0.77 |
| BURW | 0.92 | 0.54 | 0.77 | 0.76 | 0.83 | 0.51 | 0.76 | 0.75 |  |  |  |  |  |  |  |  |
| CA1 | 0.98 | 0.59 | 0.8 | 0.79 | 0.91 | 0.59 | 0.79 | 0.77 |  |  |  |  | 0.9 | 0.56 | 0.78 | 0.82 |
| CAE6 | 0.94 | 0.61 | 0.8 | 0.83 | 0.87 | 0.6 | 0.8 | 0.81 |  |  |  |  |  |  |  |  |
| CHAT | 0.92 | 0.54 | 0.77 | 0.75 |  |  |  |  |  |  |  |  |  |  |  |  |
| CHBO | 0.92 | 0.58 | 0.79 | 0.8 | 0.91 | 0.56 | 0.78 | 0.81 | 0.94 | 0.65 | 0.83 | 0.87 | 0.82 | 0.53 | 0.77 | 0.76 |
| CHBR | 0.79 | 0.57 | 0.79 | 0.76 |  |  |  |  |  |  |  |  |  |  |  |  |
| CHLG |  |  |  |  |  |  |  |  |  |  |  |  | 0.85 | 0.51 | 0.76 | 0.78 |
| CLL2 | 0.96 | 0.56 | 0.78 | 0.76 |  |  |  |  | 0.85 | 0.6 | 0.8 | 0.82 | 0.85 | 0.54 | 0.77 | 0.81 |
| COAL |  |  |  |  | 0.87 | 0.52 | 0.76 | 0.75 | 0.89 | 0.59 | 0.79 | 0.81 |  |  |  |  |
| CWMB |  |  |  |  |  |  |  |  | 0.91 | 0.55 | 0.78 | 0.78 |  |  |  |  |
| DERR | 0.83 | 0.56 | 0.78 | 0.77 |  |  |  |  | 0.98 | 0.6 | 0.8 | 0.83 |  |  |  |  |
| DESA | 0.97 | 0.59 | 0.79 | 0.81 | 0.89 | 0.58 | 0.79 | 0.79 |  |  |  |  |  |  |  |  |
| DUMB | 0.79 | 0.53 | 0.77 | 0.75 |  |  |  |  |  |  |  |  |  |  |  |  |
| EB |  |  |  |  |  |  |  |  |  |  |  |  | 0.9 | 0.53 | 0.76 | 0.76 |
| ECCL | 0.94 | 0.56 | 0.78 | 0.77 | 0.86 | 0.54 | 0.77 | 0.77 |  |  |  |  |  |  |  |  |
| ED3 | 0.89 | 0.53 | 0.77 | 0.76 |  |  |  |  | 0.94 | 0.55 | 0.77 | 0.78 |  |  |  |  |
| ESK |  |  |  |  | 0.93 | 0.52 | 0.76 | 0.8 |  |  |  |  |  |  |  |  |
| EX | 0.91 | 0.59 | 0.8 | 0.8 | 0.8 | 0.58 | 0.79 | 0.79 | 0.9 | 0.57 | 0.79 | 0.79 |  |  |  |  |
| FW |  |  |  |  |  |  |  |  | 0.92 | 0.55 | 0.78 | 0.78 |  |  |  |  |
| GGWR | 0.91 | 0.57 | 0.78 | 0.78 | 0.82 | 0.54 | 0.77 | 0.78 |  |  |  |  |  |  |  |  |
| GHSR | 0.96 | 0.59 | 0.8 | 0.81 | 0.9 | 0.57 | 0.78 | 0.79 |  |  |  |  |  |  |  |  |
| GKA8 | 0.9 | 0.6 | 0.8 | 0.8 | 0.84 | 0.62 | 0.81 | 0.82 |  |  |  |  | 0.86 | 0.49 | 0.75 | 0.77 |
| GLA4 | 0.98 | 0.55 | 0.78 | 0.76 | 0.9 | 0.59 | 0.79 | 0.79 |  |  |  |  |  |  |  |  |
| GLAZ |  |  |  |  |  |  |  |  | 0.89 | 0.65 | 0.82 | 0.86 |  |  |  |  |
| GLKP | 0.95 | 0.58 | 0.79 | 0.8 | 0.87 | 0.56 | 0.78 | 0.76 | 0.88 | 0.6 | 0.8 | 0.82 | 0.88 | 0.48 | 0.74 | 0.76 |
| GRA2 |  |  |  |  | 0.8 | 0.53 | 0.76 | 0.75 |  |  |  |  |  |  |  |  |
| HG1 | 0.98 | 0.56 | 0.78 | 0.79 | 0.94 | 0.53 | 0.77 | 0.81 |  |  |  |  |  |  |  |  |
| HG4 | 0.95 | 0.59 | 0.79 | 0.8 | 0.9 | 0.55 | 0.77 | 0.77 | 0.86 | 0.63 | 0.81 | 0.85 |  |  |  |  |
| HIL | 0.92 | 0.57 | 0.79 | 0.77 | 0.81 | 0.55 | 0.77 | 0.76 | 0.76 | 0.63 | 0.82 | 0.84 |  |  |  |  |
| HM | 0.77 | 0.53 | 0.77 | 0.77 | 0.83 | 0.51 | 0.76 | 0.75 | 0.99 | 0.6 | 0.8 | 0.84 |  |  |  |  |
| HOPE | 0.92 | 0.55 | 0.78 | 0.76 | 0.8 | 0.54 | 0.77 | 0.78 |  |  |  |  |  |  |  |  |
| HUL2 |  |  |  |  |  |  |  |  | 0.92 | 0.58 | 0.79 | 0.81 |  |  |  |  |
| HULR | 0.91 | 0.54 | 0.77 | 0.75 |  |  |  |  |  |  |  |  |  |  |  |  |
| KC1 | 0.97 | 0.59 | 0.8 | 0.79 | 0.9 | 0.54 | 0.77 | 0.78 | 0.87 | 0.64 | 0.82 | 0.86 | 0.89 | 0.55 | 0.77 | 0.83 |
| LEAM |  |  |  |  |  |  |  |  | 0.92 | 0.6 | 0.8 | 0.83 | 0.89 | 0.53 | 0.76 | 0.8 |

**Table S4 [Continued …]**

| SITE | Nitrogen Dioxide (NO_2_) | | | | Nitrogen Oxides (NOx) | | | | Ozone (O_3_) | | | | Fine Particulates (PM_2.5_) | | | |
| --- | --- | --- | --- | --- | --- | --- | --- | --- | --- | --- | --- | --- | --- | --- | --- | --- |
|  | F2 | COE | IOA | R^2^ | F2 | COE | IOA | R^2^ | F2 | COE | IOA | R^2^ | F2 | COE | IOA | R^2^ |
| LEAR | 0.91 | 0.59 | 0.8 | 0.81 | 0.88 | 0.55 | 0.77 | 0.77 |  |  |  |  | 0.91 | 0.54 | 0.77 | 0.8 |
| LECU | 0.95 | 0.56 | 0.78 | 0.77 | 0.9 | 0.54 | 0.77 | 0.75 | 0.9 | 0.61 | 0.8 | 0.83 | 0.91 | 0.53 | 0.77 | 0.79 |
| LED6 | 0.93 | 0.58 | 0.79 | 0.8 | 0.88 | 0.6 | 0.8 | 0.81 |  |  |  |  |  |  |  |  |
| LEED | 0.97 | 0.55 | 0.77 | 0.77 |  |  |  |  | 0.88 | 0.58 | 0.79 | 0.81 |  |  |  |  |
| LEIR | 0.96 | 0.58 | 0.79 | 0.79 | 0.9 | 0.58 | 0.79 | 0.78 |  |  |  |  |  |  |  |  |
| LEOM |  |  |  |  |  |  |  |  | 0.91 | 0.58 | 0.79 | 0.8 |  |  |  |  |
| LERW |  |  |  |  |  |  |  |  | 1 | 0.6 | 0.8 | 0.81 |  |  |  |  |
| LH |  |  |  |  |  |  |  |  | 0.98 | 0.56 | 0.78 | 0.79 |  |  |  |  |
| LIN3 | 0.93 | 0.59 | 0.8 | 0.79 | 0.81 | 0.61 | 0.8 | 0.8 |  |  |  |  |  |  |  |  |
| LN |  |  |  |  |  |  |  |  | 0.91 | 0.61 | 0.8 | 0.78 |  |  |  |  |
| LON6 | 0.9 | 0.57 | 0.78 | 0.77 |  |  |  |  |  |  |  |  | 0.89 | 0.53 | 0.77 | 0.81 |
| LUTR | 0.93 | 0.57 | 0.78 | 0.78 | 0.83 | 0.57 | 0.78 | 0.77 |  |  |  |  |  |  |  |  |
| LVP |  |  |  |  |  |  |  |  | 0.93 | 0.55 | 0.78 | 0.79 |  |  |  |  |
| MAHG | 0.95 | 0.54 | 0.77 | 0.75 |  |  |  |  | 0.89 | 0.62 | 0.81 | 0.84 |  |  |  |  |
| MAN3 | 0.98 | 0.56 | 0.78 | 0.79 | 0.93 | 0.55 | 0.77 | 0.77 |  |  |  |  |  |  |  |  |
| MID |  |  |  |  |  |  |  |  | 0.93 | 0.55 | 0.78 | 0.78 |  |  |  |  |
| MY1 | 0.99 | 0.57 | 0.79 | 0.79 | 0.94 | 0.62 | 0.81 | 0.81 | 0.85 | 0.6 | 0.8 | 0.81 | 0.94 | 0.53 | 0.76 | 0.8 |
| NO12 | 0.93 | 0.53 | 0.77 | 0.75 |  |  |  |  | 0.94 | 0.6 | 0.8 | 0.84 | 0.87 | 0.52 | 0.76 | 0.79 |
| NOTT | 0.98 | 0.57 | 0.78 | 0.79 | 0.93 | 0.53 | 0.76 | 0.75 | 0.89 | 0.62 | 0.81 | 0.84 | 0.88 | 0.52 | 0.76 | 0.78 |
| NWBV | 0.93 | 0.52 | 0.76 | 0.75 | 0.82 | 0.52 | 0.76 | 0.75 |  |  |  |  |  |  |  |  |
| OSY | 0.95 | 0.51 | 0.76 | 0.75 | 0.93 | 0.52 | 0.76 | 0.79 | 0.95 | 0.57 | 0.79 | 0.8 |  |  |  |  |
| OX8 |  |  |  |  |  |  |  |  |  |  |  |  | 0.89 | 0.52 | 0.76 | 0.79 |
| PEEB | 0.78 | 0.54 | 0.77 | 0.75 | 0.82 | 0.52 | 0.76 | 0.76 | 0.95 | 0.6 | 0.8 | 0.82 |  |  |  |  |
| PEMB | 0.81 | 0.55 | 0.77 | 0.79 |  |  |  |  | 0.99 | 0.62 | 0.81 | 0.84 |  |  |  |  |
| PLYM |  |  |  |  |  |  |  |  | 0.92 | 0.54 | 0.77 | 0.77 |  |  |  |  |
| PLYR | 0.89 | 0.55 | 0.78 | 0.76 |  |  |  |  |  |  |  |  |  |  |  |  |
| PRES | 0.94 | 0.56 | 0.78 | 0.78 | 0.9 | 0.52 | 0.76 | 0.75 |  |  |  |  |  |  |  |  |
| PT4 |  |  |  |  |  |  |  |  | 0.95 | 0.52 | 0.76 | 0.75 |  |  |  |  |
| REA5 | 0.9 | 0.56 | 0.78 | 0.75 |  |  |  |  |  |  |  |  |  |  |  |  |
| ROCH |  |  |  |  |  |  |  |  | 0.94 | 0.6 | 0.8 | 0.82 | 0.81 | 0.54 | 0.77 | 0.78 |
| SCN2 | 0.92 | 0.52 | 0.76 | 0.75 |  |  |  |  |  |  |  |  |  |  |  |  |
| SEND | 0.94 | 0.53 | 0.76 | 0.76 | 0.89 | 0.5 | 0.75 | 0.75 | 0.92 | 0.59 | 0.79 | 0.83 |  |  |  |  |
| SHBR | 0.95 | 0.57 | 0.78 | 0.79 | 0.88 | 0.53 | 0.77 | 0.76 |  |  |  |  |  |  |  |  |
| SHDG |  |  |  |  |  |  |  |  | 0.9 | 0.57 | 0.78 | 0.8 | 0.85 | 0.51 | 0.75 | 0.76 |
| SIB |  |  |  |  |  |  |  |  | 0.98 | 0.59 | 0.8 | 0.83 |  |  |  |  |
| SOUT |  |  |  |  |  |  |  |  | 0.88 | 0.57 | 0.79 | 0.8 |  |  |  |  |
| STKR | 0.95 | 0.58 | 0.79 | 0.79 | 0.91 | 0.59 | 0.79 | 0.79 |  |  |  |  |  |  |  |  |
| STOK | 0.95 | 0.54 | 0.77 | 0.76 |  |  |  |  | 0.92 | 0.58 | 0.79 | 0.82 |  |  |  |  |
| SUN2 | 0.86 | 0.53 | 0.76 | 0.75 |  |  |  |  | 0.95 | 0.55 | 0.78 | 0.79 |  |  |  |  |
| SUNR | 0.87 | 0.54 | 0.77 | 0.76 | 0.81 | 0.53 | 0.77 | 0.75 |  |  |  |  |  |  |  |  |
| SV |  |  |  |  |  |  |  |  | 1 | 0.63 | 0.82 | 0.85 |  |  |  |  |
| SWA1 | 0.86 | 0.54 | 0.77 | 0.75 |  |  |  |  |  |  |  |  |  |  |  |  |
| TED2 |  |  |  |  |  |  |  |  |  |  |  |  | 0.9 | 0.54 | 0.77 | 0.78 |
| TH2 | 0.98 | 0.54 | 0.77 | 0.75 | 0.94 | 0.57 | 0.79 | 0.79 |  |  |  |  |  |  |  |  |
| THUR |  |  |  |  | 0.84 | 0.49 | 0.75 | 0.78 |  |  |  |  |  |  |  |  |
| TRAN |  |  |  |  |  |  |  |  | 0.94 | 0.57 | 0.78 | 0.8 | 0.77 | 0.5 | 0.75 | 0.77 |
| WAL4 | 0.91 | 0.57 | 0.78 | 0.77 | 0.87 | 0.53 | 0.77 | 0.76 | 0.93 | 0.61 | 0.8 | 0.83 |  |  |  |  |
| WEYB |  |  |  |  |  |  |  |  | 0.98 | 0.59 | 0.79 | 0.82 |  |  |  |  |
| WFEN |  |  |  |  |  |  |  |  | 0.96 | 0.65 | 0.82 | 0.87 |  |  |  |  |
| WIG5 | 0.85 | 0.58 | 0.79 | 0.78 |  |  |  |  | 0.88 | 0.62 | 0.81 | 0.83 | 0.85 | 0.49 | 0.74 | 0.76 |
| WTHG | 0.98 | 0.57 | 0.79 | 0.8 | 0.93 | 0.6 | 0.8 | 0.79 |  |  |  |  |  |  |  |  |
| YK10 | 0.87 | 0.54 | 0.77 | 0.75 |  |  |  |  |  |  |  |  |  |  |  |  |
| YK11 | 0.94 | 0.54 | 0.77 | 0.76 | 0.88 | 0.55 | 0.78 | 0.77 |  |  |  |  | 0.86 | 0.52 | 0.76 | 0.8 |
| YW |  |  |  |  |  |  |  |  | 0.99 | 0.56 | 0.78 | 0.8 |  |  |  |  |

**Table S5:** Changes in monthly average daily traffic (ADT) counts on A-Roads and Motorways, as reported by Highways England via WebTRIS.

| **Region** | **Sites (N)** | **Vehicles** | **Monthly-average daily traffic (MADT) counts** | | | | | **% change on MADT counts recorded in 2019** | | | | |
| --- | --- | --- | --- | --- | --- | --- | --- | --- | --- | --- | --- | --- |
|  |  |  | **Jan-2020** | **Feb-2020** | **Mar-2020** | **Apr-2020** | **May-2020** | **January** | **February** | **March** | **April** | **May** |
| East Midlands | 648 | Light | 25,409 | 26,804 | 20,104 | 7,121 | 10,890 | +3.5 | +1.0 | -25.7 | -75.0 | -61.3 |
|  |  | Heavy | 6,546 | 6,738 | 6,502 | 4,415 | 4,911 | +1.8 | -0.3 | -4.2 | -34.4 | -29.0 |
|  |  | Total | 31,955 | 33,542 | 26,606 | 11,536 | 15,801 | +3.1 | +0.7 | -21.4 | -67.2 | -55.0 |
| East of England | 897 | Light | 29,842 | 30,989 | 23,022 | 8,884 | 13,747 | +1.1 | -0.7 | -27.7 | -73.2 | -58.2 |
|  |  | Heavy | 5,971 | 6,038 | 5,881 | 4,142 | 4,566 | -1.3 | -3.6 | -4.7 | -31.9 | -27.5 |
|  |  | Total | 35,813 | 37,027 | 28,903 | 13,026 | 18,313 | +0.7 | -1.2 | -24.0 | -66.8 | -53.3 |
| London | 91 | Light | 32,688 | 33,677 | 24,592 | 9,891 | 15,256 | +0.9 | -1.2 | -29.5 | -72.4 | -57.8 |
|  |  | Heavy | 6,259 | 6,364 | 6,089 | 4,117 | 4,613 | -2.4 | -5.5 | -9.6 | -35.6 | -30.2 |
|  |  | Total | 38,947 | 40,041 | 30,681 | 14,008 | 19,869 | +0.3 | -1.9 | -26.3 | -66.8 | -53.5 |
| North East | 162 | Light | 16,966 | 17,427 | 13,616 | 5,467 | 8,092 | +3.4 | -0.3 | -23.8 | -70.2 | -56.0 |
|  |  | Heavy | 1,521 | 1,576 | 1,521 | 943 | 1,078 | +1.5 | -2.7 | -6.0 | -42.6 | -35.9 |
|  |  | Total | 18,487 | 19,003 | 15,137 | 6,410 | 9,170 | +3.2 | -0.5 | -22.3 | -67.9 | -54.3 |
| North West | 698 | Light | 26,989 | 27,657 | 21,659 | 8,552 | 12,624 | +3.8 | -2.5 | -24.4 | -71.1 | -57.2 |
|  |  | Heavy | 3,908 | 4,002 | 3,894 | 2,652 | 2,930 | +2.4 | -2.6 | -5.0 | -35.2 | -30.5 |
|  |  | Total | 30,897 | 31,659 | 25,553 | 11,204 | 15,554 | +3.6 | -2.5 | -22.0 | -66.8 | -53.8 |
| South East | 1,097 | Light | 29,648 | 30,282 | 22,916 | 8,130 | 13,460 | +1.6 | -2.8 | -28.6 | -75.6 | -59.6 |
|  |  | Heavy | 3,820 | 3,937 | 3,788 | 2,440 | 2,777 | -0.7 | -4.9 | -9.7 | -39.9 | -33.9 |
|  |  | Total | 33,468 | 34,219 | 26,704 | 10,570 | 16,237 | +1.4 | -3.1 | -26.4 | -71.7 | -56.8 |
| South West | 714 | Light | 23,165 | 24,591 | 17,788 | 5,178 | 8,469 | +0.2 | -2.2 | -31.2 | -81.2 | -69.4 |
|  |  | Heavy | 3,549 | 3,686 | 3,548 | 2,360 | 2,610 | -1.6 | -3.7 | -7.9 | -39.3 | -36.4 |
|  |  | Total | 26,714 | 28,277 | 21,336 | 7,538 | 11,079 | +0.0 | -2.4 | -28.1 | -76.0 | -65.1 |
| West Midlands | 560 | Light | 22,169 | 23,016 | 17,335 | 6,778 | 10,222 | +1.8 | -1.8 | -27.3 | -72.4 | -58.2 |
|  |  | Heavy | 4,427 | 4,540 | 4,369 | 3,101 | 3,432 | -2.8 | -3.6 | -8.3 | -32.7 | -28.1 |
|  |  | Total | 26,596 | 27,556 | 21,704 | 9,879 | 13,654 | +1.0 | -2.1 | -24.2 | -66.1 | -53.3 |
| Yorkshire & The Humber | 1,031 | Light | 24,779 | 25,693 | 19,818 | 7,315 | 10,965 | +2.5 | -2.0 | -25.1 | -73.3 | -59.5 |
|  |  | Heavy | 5,336 | 5,511 | 5,386 | 3,671 | 4,037 | -0.6 | -2.9 | -5.0 | -34.2 | -28.9 |
|  |  | Total | 30,115 | 31,204 | 25,204 | 10,986 | 15,002 | 2.0 | -2.1 | -21.6 | -66.7 | -54.2 |
| England (All Regions) | 5,898 | Light | 26,250 | 27,215 | 20,552 | 7,510 | 11,654 | +2.0 | -1.7 | -27.2 | -74.4 | -60.2 |
|  |  | Heavy | 4,721 | 4,847 | 4,696 | 3,194 | 3,546 | -0.4 | -3.1 | -6.2 | -35.2 | -30.3 |
|  |  | Total | 30,971 | 32,062 | 25,248 | 10,704 | 15,200 | +1.6 | -1.9 | -24.0 | -68.8 | -55.7 |

**Table S6:** Selection of weekly-average meteorological profiles at urban background sites across the UK, for weeks 14 to 18 of 2020. Wind direction is calculated as a scalar average.

| Location | Week | Start date | End date | Wind speed (m/s) | Wind direction (°) | Air temperature | |
| --- | --- | --- | --- | --- | --- | --- | --- |
|  |  |  |  |  |  | Daily-maximum (°C) | % change on expected **†** |
| Cardiff Centre  (Wales) | 14 | 30/03/2020 | 05/04/2020 | 4.9 | 50.4 | 11.7 | + 1.4 |
|  | 15 | 06/04/2020 | 12/04/2020 | 3.5 | 54.6 | 18.8 | + 63.5 |
|  | 16 | 13/04/2020 | 19/04/2020 | 5.5 | 72.6 | 14.3 | + 24.2 |
|  | 17 | 20/04/2020 | 26/04/2020 | 4.9 | 65.5 | 18.7 | + 62.9 |
|  | 18 | 27/04/2020 | 03/05/2020 | 4.8 | 259.5 | 12.1 | + 5.0 |
| Edinburgh St Leonards  (Scotland) | 14 | 30/03/2020 | 05/04/2020 | 5.8 | 266.7 | 11.0 | - 4.2 |
|  | 15 | 06/04/2020 | 12/04/2020 | 4.9 | 248.4 | 13.2 | +14.6 |
|  | 16 | 13/04/2020 | 19/04/2020 | 4.9 | 55.3 | 10.6 | - 7.8 |
|  | 17 | 20/04/2020 | 26/04/2020 | 4.1 | 55.5 | 11.7 | + 1.4 |
|  | 18 | 27/04/2020 | 03/05/2020 | 3.6 | 57.9 | 10.7 | - 6.8 |
| London North Kensington  (London) | 14 | 30/03/2020 | 05/04/2020 | 3.0 | 1.0 | 13.3 | + 16.0 |
|  | 15 | 06/04/2020 | 12/04/2020 | 2.5 | 112.7 | 21.7 | + 88.6 |
|  | 16 | 13/04/2020 | 19/04/2020 | 3.4 | 73.0 | 15.5 | + 35.2 |
|  | 17 | 20/04/2020 | 26/04/2020 | 3.4 | 75.1 | 19.8 | + 71.8 |
|  | 18 | 27/04/2020 | 03/05/2020 | 3.8 | 251.6 | 13.0 | + 13.4 |
| Leicester University  (East Midlands) | 14 | 30/03/2020 | 05/04/2020 | 3.8 | 269.5 | 12.2 | + 5.7 |
|  | 15 | 06/04/2020 | 12/04/2020 | 2.7 | 138.1 | 19.5 | + 69.2 |
|  | 16 | 13/04/2020 | 19/04/2020 | 3.5 | 76.7 | 13.6 | + 18.2 |
|  | 17 | 20/04/2020 | 26/04/2020 | 3.6 | 77.1 | 17.2 | + 49.8 |
|  | 18 | 27/04/2020 | 03/05/2020 | 3.7 | 242.0 | 11.2 | - 2.2 |

**†** *Weekly average daily-maximum air temperatures are compared to the long-term (1981-2010) mean daily-maximum air temperature for the month of April, which the Met Office reports as 11.5°C.*

**Table S7:** Regional summary of urban background sites in England, Scotland, Wales, and Northern Ireland.

| Site Type | 5-week average daily concentrations,  covering April 2020 in µg/m^3^ (AURN count) **†** | | | | % change on the 5-week average daily concentration measured in 2017-19 **†** | | | | Absolute change on the 5-week average daily concentration measured in 2017-19 (µg/m^3^) **†** | | | | % days below the 5-week average daily concentration in 2017-19 | | | |  |
| --- | --- | --- | --- | --- | --- | --- | --- | --- | --- | --- | --- | --- | --- | --- | --- | --- | --- |
|  | NO_2_ | NO_X_ | O_3_ | PM_2.5_ | NO_2_ | NO_X_ | O_3_ | PM_2.5_ | NO_2_ | NO_X_ | O_3_ | PM_2.5_ | NO_2_ | NO_X_ | O_3_ | PM_2.5_ | |
| North West | 12.0 (6) | 15.1 (6) | 71.1 (3) | 10.4 (6) | -37.4 | -38.8 | +4.3 | -14.1 | -7.2 | -9.6 | +2.9 | -1.7 | 89.0 | 88.5 | 36.2 | 69.0 | |
| North East | 9.7 (1) | 13.6 (1) | 69.4 (1) | - | -14.0 | -22.9 | -0.5 | - | -1.6 | -4.0 | -0.3 | - | 57.1 | 71.4 | 57.1 | - | |
| Yorkshire & The Humber | 13.5 (6) | 17.0 (6) | 65.8 (4) | 10.2 (4) | -30.1 | -34.9 | +9.8 | -23.7 | -5.8 | -9.1 | +5.9 | -3.2 | 82.4 | 86.2 | 30.7 | 77.6 | |
| West Midlands | 11.6 (4) | 14.5 (4) | 68.8 (5) | 9.2 (1) | -38.2 | -42.2 | +12.0 | -33.7 | -7.2 | -10.6 | +7.4 | -4.7 | 87.1 | 91.4 | 30.3 | 85.7 | |
| East Midlands | 13.0 (2) | 16.8 (2) | 67.5 (2) | 9.7 (3) | -45.9 | -48.1 | +18.2 | -25.7 | -11.0 | -15.6 | +10.4 | -3.4 | 91.4 | 92.9 | 17.1 | 80.0 | |
| East of England | 12.8 (3) | 16.4 (3) | 67.7 (2) | 10.5 (1) | -28.8 | -28.2 | +10.8 | -29.8 | -5.2 | -6.4 | +6.6 | -4.5 | 79.0 | 79.0 | 22.9 | 85.7 | |
| South West | 11.5 (4) | 20.5 (4) | 70.9 (3) | 15.6 (2) | -20.8 | +1.0 | +10.2 | +16.3 | -3.0 | +0.2 | +6.6 | 2.2 | 72.6 | 72.1 | 24.8 | 52.9 | |
| South East | 14.4 (4) | 19.3 (4) | 62.7 (1) | 13.3 (3) | -25.3 | -25.3 | +16.4 | -14.7 | -4.9 | -6.5 | +8.8 | -2.3 | 76.4 | 77.1 | 25.7 | 72.4 | |
| London | 21.1 (4) | 26.1 (4) | 61.6 (4) | 13.0 (3) | -38.0 | -42.9 | +23.0 | -12.6 | -12.9 | -19.6 | +11.5 | -1.9 | 85.0 | 87.1 | 20.0 | 72.4 | |
| Scotland | 8.0 (4) | 10.2 (4) | 59.9 (4) | 6.5 (2) | -44.7 | -42.5 | -11.0 | -26.5 | -6.5 | -7.5 | -7.4 | -2.3 | 95.0 | 95.7 | 62.1 | 88.6 | |
| Wales | 8.7 (1) | 11.2 (1) | 71.6 (1) | 11.7 (1) | -56.6 | -55.1 | +6.6 | -11.6 | -11.3 | -13.7 | +4.4 | -1.5 | 100.0 | 100.0 | 34.3 | 65.7 | |
| Northern Ireland | 6.5 (2) | 8.8 (2) | 65.3 (1) | 8.5 (2) | -44.8 | -41.6 | -5.6 | -28.2 | -5.3 | -6.3 | -3.9 | -3.3 | 88.6 | 85.7 | 62.9 | 78.6 | |
| United Kingdom (All Regions) | 12.6 (41) | 16.6 (41) | 66.4 (31) | 10.9 (28) | -36.7 | -37.8 | +7.4 | -17.3 | -7.3 | -10.1 | 4.6 | -2.3 | 84.1 | 85.6 | 33.7 | 74.2 | |

**†** *Calculations required at least 95% of the daily-average measurements to be available in (a) weeks 14 to 18 of 2020, and (b) weeks 14 to 18 of 2017-19. Days were excluded from the 5-week average calculations, where less than 18-hours of measurement data is available.*

**Table S8**: Regional summary of urban traffic sites in England, Scotland, Wales, and Northern Ireland.

| Site Type | 5-week average daily concentrations,  covering April 2020 in µg/m^3^ (AURN count) **†** | | | | % change on the 5-week average daily concentration measured in 2017-19 **†** | | | | Absolute change on the 5-week average daily concentration measured in 2017-19 (µg/m^3^) **†** | | | | % days below the 5-week average daily concentration in 2017-19 | | | |
| --- | --- | --- | --- | --- | --- | --- | --- | --- | --- | --- | --- | --- | --- | --- | --- | --- |
|  | NO_2_ | NO_X_ | O_3_ | PM_2.5_ | NO_2_ | NO_X_ | O_3_ | PM_2.5_ | NO_2_ | NO_X_ | O_3_ | PM_2.5_ | NO_2_ | NO_X_ | O_3_ | PM_2.5_ |
| North West | 14.1 (3) | 21.9 (3) | - | 10.7 (1) | -41.0 | -43.9 | - | -7.0 | -9.8 | -17.1 | - | -0.8 | 91.4 | 92.4 | - | 71.4 |
| North East | 8.5 (3) | 12.6 (3) | - | 9.1 (1) | -48.0 | -49.1 | - | -18.0 | -7.8 | -12.2 | - | -2.0 | 96.2 | 95.2 | - | 77.1 |
| Yorkshire & The Humber | 17.3 (6) | 30.5 (6) | - | 8.7 (1) | -48.8 | -55.3 | - | -31.0 | -16.5 | -37.7 | - | -3.9 | 96.2 | 95.7 | - | 77.1 |
| West Midlands | 20.3 (2) | 39.1 (2) | 62.4 (1) | 10.0 (1) | -37.6 | -41.1 | +20.9 | -26.4 | -12.2 | -27.3 | +10.8 | -3.6 | 85.7 | 87.1 | 20.0 | 85.7 |
| East Midlands | 16.0 (4) | 28.2 (4) | - | 9.7 (1) | -53.6 | -61.6 | - | -22.3 | -18.5 | -45.2 | - | -2.8 | 98.6 | 99.3 | - | 77.1 |
| East of England | 24.5 (2) | 47.3 (2) | - | - | -34.4 | -38.0 | - | - | -12.8 | -29.0 | - | - | 82.9 | 85.7 | - | - |
| South West | 16.8 (4) | 25.3 (4) | 58.0 (1) | 13.9 (1) | -40.7 | -50.7 | +20.3 | +23.2 | -11.5 | -26.0 | +9.8 | 2.6 | 92.0 | 94.9 | 14.3 | 54.3 |
| South East | 20.5 (5) | 34.6 (5) | - | - | -36.6 | -46.5 | - | - | -11.8 | -30.1 | - | - | 86.9 | 93.7 | - | - |
| London | 25.9 (4) | 38.2 (4) | 53.0 (1) | 13.3 (2) | -49.9 | -63.7 | +79.5 | -25.5 | -25.8 | -67.0 | +23.5 | -4.6 | 97.9 | 99.3 | - | 75.7 |
| Scotland | 13.3 (9) | 22.8 (9) | - | 6.2 (2) | -58.3 | -64.3 | - | -30.3 | -18.6 | -41.1 | - | -2.7 | 100.0 | 100.0 | 0.0 | 90.0 |
| Wales | 20.6 (3) | 40.1 (3) | - | 13.6 (1) | -41.1 | -49.8 | - | -1.7 | -14.4 | -39.8 | - | -0.2 | 94.3 | 98.1 | - | 60.0 |
| Northern Ireland | 22.4 (2) | 43.8 (2) | - | - | -24.3 | -38.5 | - | - | -7.2 | -27.4 | - | - | 74.3 | 87.1 | - | - |
| United Kingdom (All Regions) | 17.5 (47) | 30.0 (47) | 57.8 (3) | 10.4 (11) | -47.9 | -57.3 | +34.1 | -18.1 | -16.1 | -40.3 | +14.7 | -2.3 | 93.5 | 95.6 | 11.4 | 75.8 |

**†** *Calculations required at least 95% of the daily-average measurements to be available in (a) weeks 14 to 18 of 2020, and (b) weeks 14 to 18 of 2017-19. Days were excluded from the 5-week average calculations, where less than 18-hours of measurement data is available.*

**Table S9:** UK Automatic Hydrocarbon Network provisionally verified summaries of benzene (B), toluene (T), ethylbenzene (E), and o-xylene (X).

| UK Automatic Hydrocarbon Network | | 5-week average daily concentrations,  covering April 2020 (µg/m^3^) **†** | | | | % change on the 5-week average daily concentration measured in 2017-19 **†** | | | | % days below the 5-week average daily concentration in 2017-19 | | | | |
| --- | --- | --- | --- | --- | --- | --- | --- | --- | --- | --- | --- | --- | --- | --- |
| Site Name | Type ***** | B | T | E | X | B | T | E | X | B | T | E | X |  |
| Auchencorth Moss | RB | - | - | - | - | - | - | - | - | - | - | - | - |  |
| Chilbolton Observatory | RB | - | 0.53 | 0.12 | 0.12 | - | +25.53 | +25.70 | +25.55 | - | 43.8 | 37.5 | 40.6 |  |
| London Eltham | SB | 0.34 | 0.50 | 0.13 | 0.12 | -18.74 | -27.93 | -18.66 | -28.51 | 81.3 | 84.4 | 77.4 | 80.6 |  |
| London Marylebone Road | UT | 0.58 | 1.46 | 0.37 | 0.46 | -25.79 | -33.36 | -14.27 | -15.62 | 89.3 | 85.7 | 82.1 | 82.1 |  |

***** *RB = Rural background, SB = Suburban background, UT = Urban traffic*

**†** *Calculations required at least 75% of the daily-average measurements to be available in (a) weeks 14 to 18 of 2020, and (b) weeks 14 to 18 of 2017-19. Days were excluded from the 5-week average calculations, where less than 18-hours of measurement data is available.*

**Table S10:** The average relative influence of each variable on UK pollution concentrations, as reported by boosted regression tree (BRT) models of AURN sites with a cross-validation r-squared ≥ 0.75.

| Modelled Influence | | Relative Influence (%) | | | |
| --- | --- | --- | --- | --- | --- |
|  |  | Nitrogen Dioxide (NO_2_) | Nitrogen Oxides (NOx) | Ozone (O_3_) | Fine Particulates (PM_2.5_) |
| Temporal | Trend | 9.5 | 11.3 | 10.0 | 30.0 |
|  | Season | 3.2 | 3.2 | 8.9 | 1.1 |
|  | Week | 5.0 | 4.7 | 7.6 | 11.3 |
|  | Weekday | 4.1 | 4.5 | 1.4 | 2.0 |
|  | Hour | 19.5 | 18.1 | 3.5 | 2.8 |
|  | Total | 41.3 | 41.8 | 31.4 | 47.2 |
| Meteorological | Wind Speed | 16.2 | 14.7 | 14.0 | 9.1 |
|  | Wind Direction | 14.5 | 11.1 | 9.9 | 16.4 |
|  | Air Temperature | 13.6 | 14.6 | 7.8 | 5.6 |
|  | Atmospheric Pressure | 3.8 | 5.3 | 4.3 | 6.8 |
|  | Dew Point | 3.4 | 3.0 | 3.0 | 5.3 |
|  | Relative Humidity | 4.2 | 5.8 | 27.4 | 4.7 |
|  | Cloud Ceiling Height | 3.0 | 3.7 | 2.2 | 4.9 |
|  | Total | 58.7 | 58.2 | 68.6 | 52.8 |
| Total | | 100.0 | 100.0 | 100.0 | 100.0 |

**Figure S1:** Map of the United Kingdom detailing the Government Office Regions (GOR), excluding Northern Ireland.

**
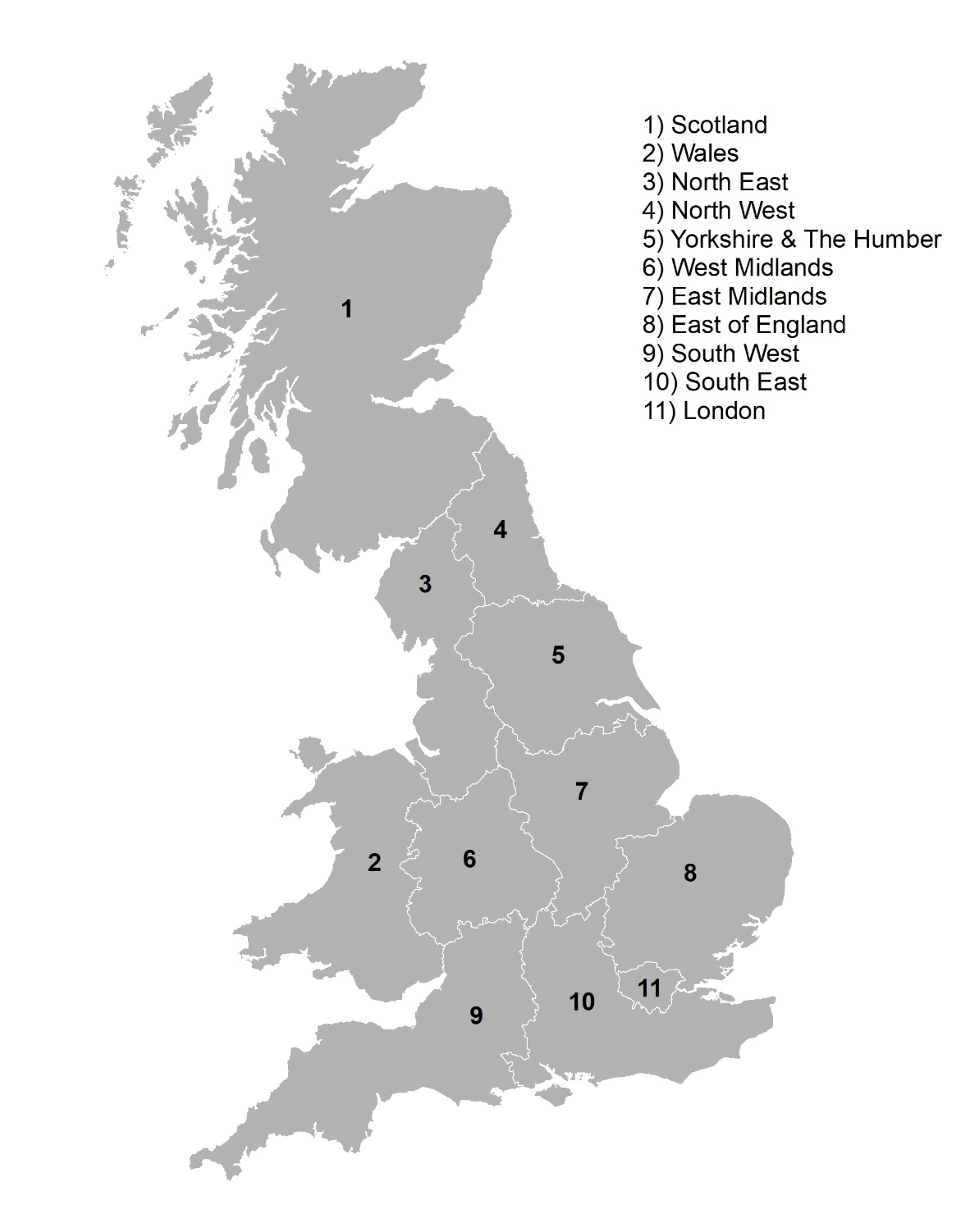
**

**Figure S2:** Regional summary of percentage changes in NO_2_ and PM_2.5_ concentration across traffic and background AURN sites.

**
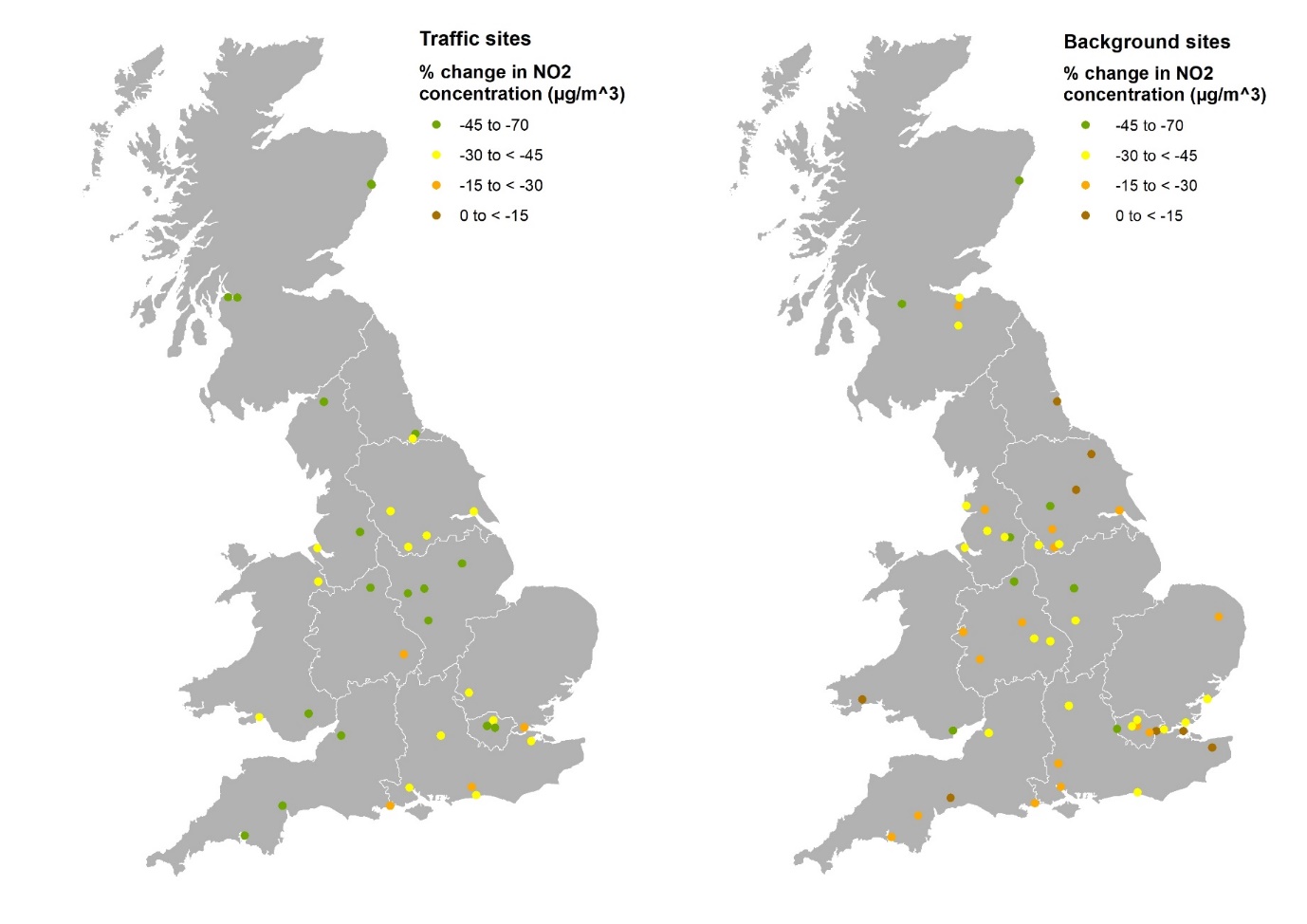
**

**
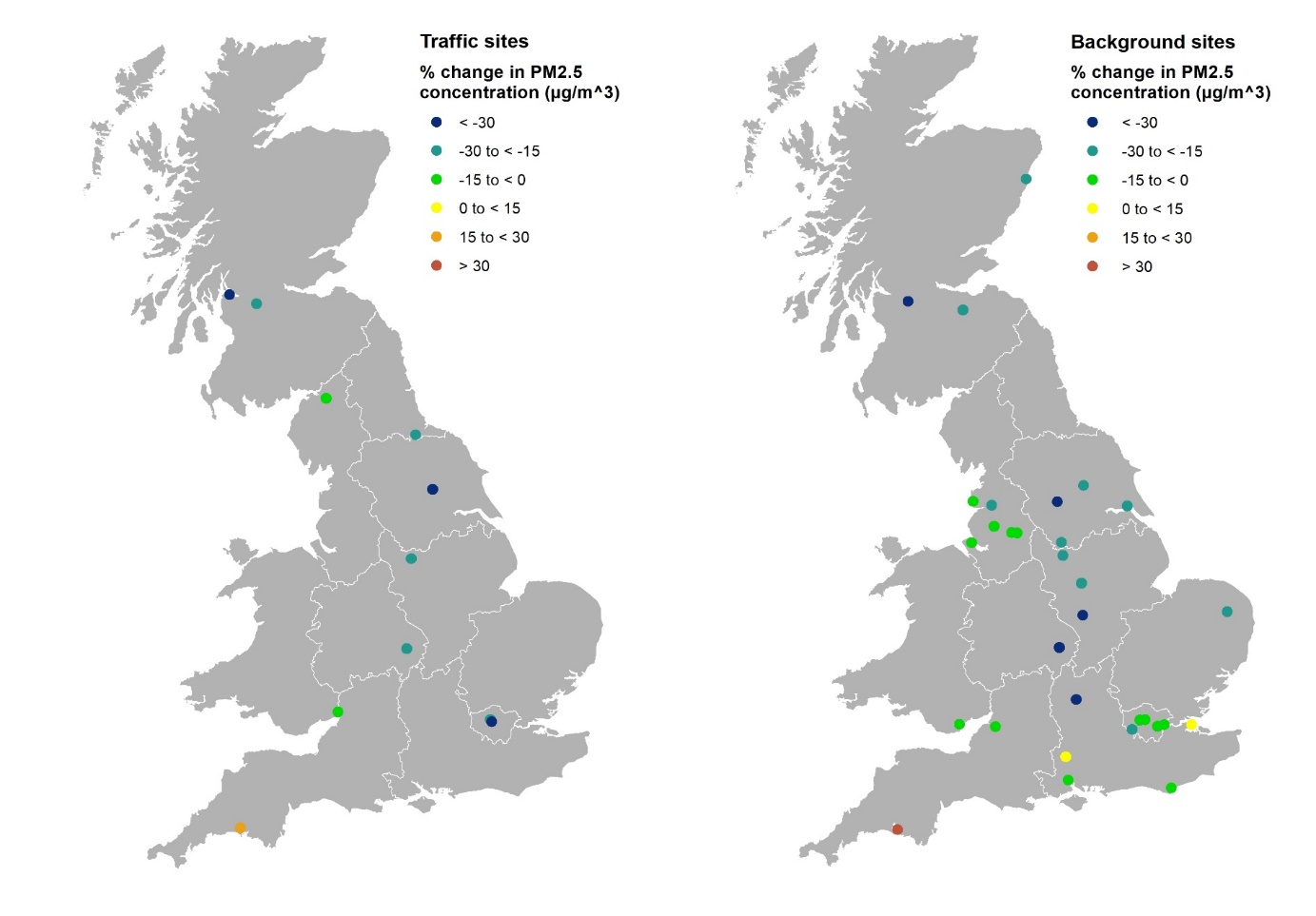
**

**References**

Carslaw, D.C. and Taylor, P.J., 2009. Analysis of air pollution data at a mixed source location using boosted regression trees. Atmospheric Environment, 43(22 - 23), pp. 3563 - 3570.

Carslaw, D.C., Williams, M.L. and Barratt, B., 2012. A short-term intervention study—Impact of airport closure due to the eruption of Eyjafjallajökull on near-field air quality. Atmospheric environment, Volume 54, pp. 328 - 336.

DEFRA, 2020. UK Air - Air Information Resource (Online). Available from: https://uk-air.defra.gov.uk/networks/site-types [Accessed 2020].

Elith, J., Leathwick, J.R. and Hastie, T., 2008. A working guide to boosted regression trees. Journal of Animal Ecology, 77(4), pp. 802 - 813.

Friedman, J., 1999. Greedy function approximation: a gradient boosting machine. Annals of statistics, pp. 1189 - 1232.

Hastie, T., Tibshirani, R. and Friedman, J., 2001. The elements of statistical learning.

Ibrahim, Z., Yahaya, N., and Yahaya, J., 2019. Use of the Boosted Regression Tree Optimization Technique to Analyse Air Pollution data. International Journal of Recent Technology and Engineering, 8(4), pp.1565-1575
